# Beyond bariatric surgery and weight loss medicaments. A systematic review of the current practice in obesity rehabilitative inpatient programs in adults and pediatrics

**DOI:** 10.3389/fnut.2022.963709

**Published:** 2022-09-29

**Authors:** Daniele Spadaccini, Silvia Guazzotti, Filipa Patricia Goncalves Correia, Tommaso Daffara, Sabrina Tini, Alessandro Antonioli, Gianluca Aimaretti, Paolo Marzullo, Marina Caputo, Valentina Antoniotti, Flavia Prodam

**Affiliations:** ^1^Department of Health Sciences, University of Piemonte Orientale, Novara, Italy; ^2^Biological Mass Spectrometry Lab, Department of Translational Medicine, University of Piemonte Orientale, Novara, Italy; ^3^Endocrinology, Department of Translational Medicine, University of Piemonte Orientale, Novara, Italy; ^4^Division of General Medicine, IRCCS Istituto Auxologico Italiano, Ospedale San Giuseppe, Verbania, Italy; ^5^SCDU of Pediatrics, Department of Health Sciences, University of Piemonte Orientale, Novara, Italy

**Keywords:** obesity, treatment, prevention, inpatient setting, weight loss

## Abstract

**Background:**

Obesity treatment strategies mainly include outpatient lifestyle modification, drugs and bariatric surgery. Voluntary rehabilitative inpatient programs are gaining relevance as potential alternative settings of care that focus on weight loss and prevention of weight regain through a multidisciplinary approach, but their prevalence is still limited due to the high costs.

**Aim:**

Considering the lack of evidence in this area, the objective of this study is to systematically review the currently available literature on non-pharmacological and non-surgical inpatient programs aimed at weight loss, to clarify the efficacy and the characteristics of these interventions.

**Methods:**

Proper English language articles from 2000 to 2022 were searched on relevant databases. Quality assessment was performed by two different authors using *ROB2* and *robvis* tools. Adult and pediatric studies were reviewed separately and their characteristics were systematically displayed.

**Results:**

36 articles were included (20 on adults, 16 on children, and adolescents) for a total of 5,510 individuals. The multidisciplinary approach was mainly comprehensive of a low-calorie diet, scheduled physical activity, and psychological support based on behavioral treatment. Educational and cooking sessions were present at a lower rate. Globally, inpatient weight loss programs showed a consistent efficacy in reducing body weight and inducing beneficial effects on quality of life, psychological well-being, eating behavior, physical performance, and fatigue. Follow-up data were scarce, but with a high percentage of patients regaining weight after a short period.

**Conclusion:**

Weight loss inpatient rehabilitation is a promising area that has evidence of all-rounded success in the amelioration of several aspects related to obesity. Nevertheless, it appears to be quite inconsistent in preserving these benefits after the intervention. This might slow the innovation process in this area and preclude further investments from national healthcare. Personalized and enriched programs could show greater impact when focusing on the behavioral and educational aspects, which are crucial points, in particular in pediatrics, for setting up a long-lasting lifestyle modification. More studies are therefore necessary to evaluate long-term efficacy based on the different work-up models.

## Introduction

Obesity is a morbid condition characterized by excessive adipose tissue accumulation and an increased risk of diseases such as type 2 diabetes, cancer, cardiovascular disease, musculoskeletal disease, and depression, enhancing the incidence of disability and mortality (1). Recently, both European and American Endocrinologist Associations found an agreement in determining a new diagnostic term, Adiposity-Based Chronic Disease (ABCD), which carries the significance of a widely recognized disease by contemporarily focusing the attention on the actual key elements to further the care of patients (2, 3). Despite this latest shift in the paradigm, the prevalence of obesity has still been dramatically increasing worldwide becoming a relevant problem for national care systems (4).

When treating people with obesity, carefully selecting evidence-based interventions is a crucial point. Current opinion on obesity treatment has been developing asymmetrically between countries throughout the years, with different cut-offs and recommended interventions in each case, gradually leading clinicians to challenging situations when deciding which guideline to follow. Even though both drugs and bariatric surgery are not to be considered long-term resolution interventions for weight management, they are widely suggested, especially in adults, often without a precise assessment of previous attempts of losing weight with a lifestyle modification, or a complete psychiatric evaluation (5–7).

Lifestyle intervention is agreed to be the only sustainable intervention that could be continued lifelong and should be addressed especially in the early stages of private or public health care preferably by a multidisciplinary team comprised of several figures such as physicians, medical doctors, nutrition specialists, and psychologists (4). Nevertheless, in most national and international guidelines, a difference between outpatient and inpatient lifestyle interventions is frequently overlooked and nutritional rehabilitation in inpatient settings is only provided when obese people are already hospitalized for chronic or post-acute illnesses, even if obesity is not the main cause (8, 9). However, the level of agreement among evidence-based guidelines on how to best manage inpatient weight loss rehabilitation programs is completely unclear.

European Association for the Study of Obesity (EASO) emphasizes the importance of a comprehensive approach to obesity management, which includes a wide spectrum of evidence-based treatment options, such as calorie-controlled diets, physical activity counseling, nutritional advice including structured intensive supervision, cognitive-behavioral therapy (CBT) and, in special cases, formula diets and other food replacement (10). Following these recommendations, and due to an upgrade of national guidelines in some countries, a gradual rise in the suggestion of obesity rehabilitation programs, which apply the same theory of a multidisciplinary intervention, but with a shift toward an inpatient setting, has been observed.

In Italy, the latest national SIO-ADI guidelines define a specific third level of interventions for people suffering from severe obesity with a BMI equal to or greater than 40 kg/m^2^, or than 35 kg/m^2^ in association with comorbidities (11). This intervention occurs in patients who had failed previously dietary and pharmacological treatments and is performed in the context of a semi-residential or residential facility equipped with departments of dietetics, clinical nutrition, and endocrinology under the presence of a multidisciplinary medical health team. In Germany, weight-loss programs should be individualized and approved only if their success has been shown by clinical trials (12), while in Scotland dietary interventions for weight loss should be calculated to produce a 600 kcal/day energy deficit, based on individual preferences (13). In other countries lacking specific regulation, inpatient obesity rehabilitation programs are currently promoted by private care initiatives and the treatment ranges from a few weeks to several months. Outside Europe, it does not exist any written evidence of the applicability of inpatient weight loss programs as a tool to face obesity, unless it is necessary for the procedures before and after bariatric surgery (11, 14).

In pediatrics, several societies claim that weight management work-up in the home setting is among the most efficacious in reducing BMI z-score. Physical, psychological, and behavioral therapies are regarded as critical components of childhood obesity management and should be provided by a well-trained multidisciplinary team (15, 16). Few countries in Europe, including Switzerland and Belgium, have recognized childhood obesity as a chronic disease, and multidisciplinary therapeutic programs have been developed and disseminated on a national scale, also in inpatient settings (17, 18). Compared to adults, it appears that pediatric obesity could also be addressed, by primary care settings, to reduce pressure on secondary care hospitals and prevent comorbidities at earlier stages (19).

Based on current evidence, we reviewed the most recent studies on voluntary rehabilitation in hospital settings aimed at weight loss without pharmacological, or bariatric surgery treatments, in order to clarify the role and the efficacy of this approach in the short term and the differences in the work-up models.

## Materials and methods

### General aspects

This systematic review was conducted in observance of the requirements of the latest PRISMA guidelines (PRISMA 2020 Checklist) and the protocol was written according to the Preferred Reporting Items for Systematic Reviews and Meta-Analyses (PRISMA) statement (20).

### Search criteria

Studies written in English were searched within the period 2000–2022 on Pubmed Central, Pubmed, Medline, and EMBASE databases. The research was conducted by two authors (DS and SG), using the following keyword strings: (“obesity “OR” overweight “) AND (“hospitalized “OR” inpatient “) AND (“multidisciplinary” “OR” diet “OR” nutrition “OR” “Intervention” OR “weight” OR “rehabilitation”). No country restrictions were imposed. Studies before the year 2000 were excluded since they are not indicative of current clinical practice. At a later stage, if the inclusion criteria were met, further articles taken from the bibliographies of the cited studies were considered.

### Inclusion and exclusion criteria

**FIGURE 1:**
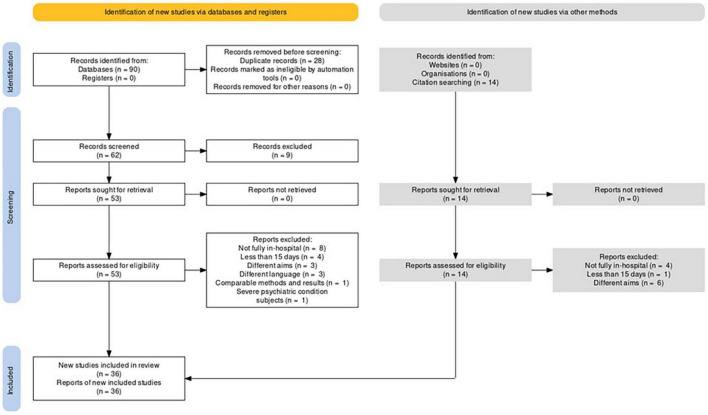
Flow chart according to PRISMA 2020 standards.

The protocol of selection was arranged to include non-pharmacological and non-surgical interventions, aimed at weight loss in residential care, with or without dietary intervention in any age, including children and adolescents, and to exclude studies that (i) provided for a treatment that was not completely carried out in the context of hospitalization; (ii) lasted less than 15 days; (iii) did not aim at weight loss; (iv) utilized weight management drugs or bariatric surgery; (v) treated the same patients of another study, with overlapping methods of intervention and results; (vi) had abstracts in English and body in another language; (vii) were in the form of abstract, letter to editor, case report, case series, or review. A flow chart following PRISMA 2020 diagram standards has also been arranged (Figure 1).

### Data extraction and synthesis

The characteristics and the most important information of the single studies were collected by three different authors (DS, SG, and VA) and by separating data depending on the age of the subjects (adults and minors). The following aspects were then extracted: author, year of publication, country, and place where the study was conducted, inclusion, and exclusion criteria, type of dietary treatment, types of non-dietary treatment, duration of treatment, outcomes of interest, type of randomization, presence of a control group, and a summary of conclusions. The information obtained from the studies was classified and represented in the form of simple absolute or relative frequencies.

### Quality assessment and risk of bias

The quality of the studies was separately conducted by two authors (DS and VA) and assessed by applying, for non-randomized longitudinal studies, the ROBINS-I (“Risk Of Bias In Non-randomized Studies of Interventions”) tool (21), which covers the following bias: confounding; selection of participants; classification of interventions; deviations from intervention; missing data; outcome measurement; and selection of the results. The categories for domain-level judgments were: low, moderate, serious, critical risk, and no information. The study was classified as low or moderate risk if it was judged to be at low or moderate risk of bias for all domains, at serious risk if at least one domain showed a serious risk of bias and the same concept for critical risk.

The risk of bias within the included Randomized Controlled Trials (RCTs) was assessed using the Cochrane’s revised risk of bias tool RoB 2, also including the supplementary tool for crossover randomized studies (22). The following biases were considered: randomization process; deviations from interventions; missing outcome data; outcome measurement; and selection of the results. Each domain was judged: low or high risk, and some concerns. The study was classified as low risk if a low risk of bias for all domains was demonstrated, and a high risk of bias if high risk in at least one domain, or concerns in multiple domains were demonstrated. If the study was judged to have some concerns in at least one domain but without at high risk of bias for any domain, it was classified as having some concerns (22). Bias results were represented by using the web app *robvis* (23). Disagreements were resolved by discussion between the reviewers, after reading the articles again. K for agreement between reviewers was 90% (k Cohen: 0.901) after screening titles and abstracts, 94% after screening full-text articles and 100% after subsequent discussions.

## Results

Our search identified a total of 90 potentially eligible articles, (Figure 1). Of these, 28 were excluded as duplicates. The remaining 62 records were screened by reading abstracts and nine studies were excluded as the model of the study was not compatible with the review. A total of 53 articles were full-text evaluated, to which 14 articles found via citation searching have been added. Among these, 31 were excluded according to the criteria imposed. 36 articles were found to be valid and included in the review, dividing them into adult (*n* = 20) and pediatric studies (*n* = 16).

### Description of studies

#### Adults

The main characteristics of the studies are summarized in Supplementary Tables 1, 2.

The 20 studies in adults included a total of 2,030 individuals. Approximately 65% of the participating subjects were women. The Italian studies were 50% of the total (24–33), while the other weight reduction programs were conducted in the USA (34–36), Norway (37, 38), Germany (39, 40), Czechia (41), Brasil (42), and Taiwan (43).

The setting was named differently, such as “weight disorder inpatient unit,” “rehabilitation clinic,” or “university hospital.”. The inclusion and exclusion criteria heavily depended on the context, in particular, studies that took into consideration routine treatments had less specific criteria. Age was chosen as a cut-off for inclusion only in three studies (27, 33, 35) and three studies recruited only women (33, 34, 41). The study design was largely dependent on its aim. Studies that were more oriented on experimentation preferred to use a two-arm randomized controlled design (25, 32, 35, 40, 43); however, most of the papers involved non-randomized participants, with a high prevalence of longitudinal studies (24, 26, 27, 29, 30, 34, 36, 37, 39, 41, 42) of which only three with a control arm (28, 31, 38). Only one study applied a randomized crossover design (33). Dietary interventions were almost entirely consisting of a low-calorie diet, except for two studies (35, 40) in which the diet administered was normocaloric and weight loss was achieved exclusively by increasing physical activity. The latter was contemplated in many, but not in all studies (32–34, 36, 42). The aerobic activity turned out to be more frequently applied than anaerobic activity, although both were often used. Other identified multidisciplinary interventions were psychotherapeutic treatment, present in about half of the studies almost always with a cognitive-behavioral approach, both for individuals and for groups (24, 25, 27, 29–32, 38–41), nutritional education (25, 26, 29–31, 37–40) and healthy cooking lessons (37). Follow-up data (up to 2 years) were present only in three studies (25, 38, 40).

#### Children and adolescents

The studies included were 16 for a total of 3,480 individuals. Also in this case, the female gender was more prevalent, equal to about 60% of the whole sample. The country with the highest number of articles emerged to be Germany (44–48), followed by Switzerland (49–51), Belgium (52–54), Italy (55, 56), France (57, 58), and the Czechia (59). As for studies in adults, the setting description was heterogeneous but the presence of a specialized pediatric department was constant. Criteria of inclusion for the BMI were generally quite specific but variable among studies; in particular, 14 studies applied BMI cutoffs based on SD or percentiles (44–49, 52–56, 58, 59), one chose IOTF standards (57), and two studies on adolescents preferred the adult BMI cut-offs (50, 51). Age ranged between 6 and 19 years. Among exclusion criteria, syndromic and secondary obesity were the most frequently declared (44–51, 54, 58). Most of the studies applied a single-arm open-label design (44, 45, 48–55, 58, 59) with the remaining ones being non-randomized interventions against control or comparable groups (46, 47, 56); only one study was an RCT (57). The prevailing dietary treatment was a balanced low-calorie diet, although in certain studies an *ad libitum* buffet approach (46, 57) or a normocaloric diet accompanied by physical activity were used (53, 58). Physical activity was part of the multidisciplinary treatment in all studies, while psychotherapy, generally with a behavioral approach, was provided for less than half of the interventions (44, 46–48, 52, 54, 58). Nutritional education was also present in five studies (44, 46–48, 58). Three studies reported follow-up data up to 14 months after the intervention (46, 48, 52).

### Risk of bias within non-randomized longitudinal studies

**FIGURE 2:**
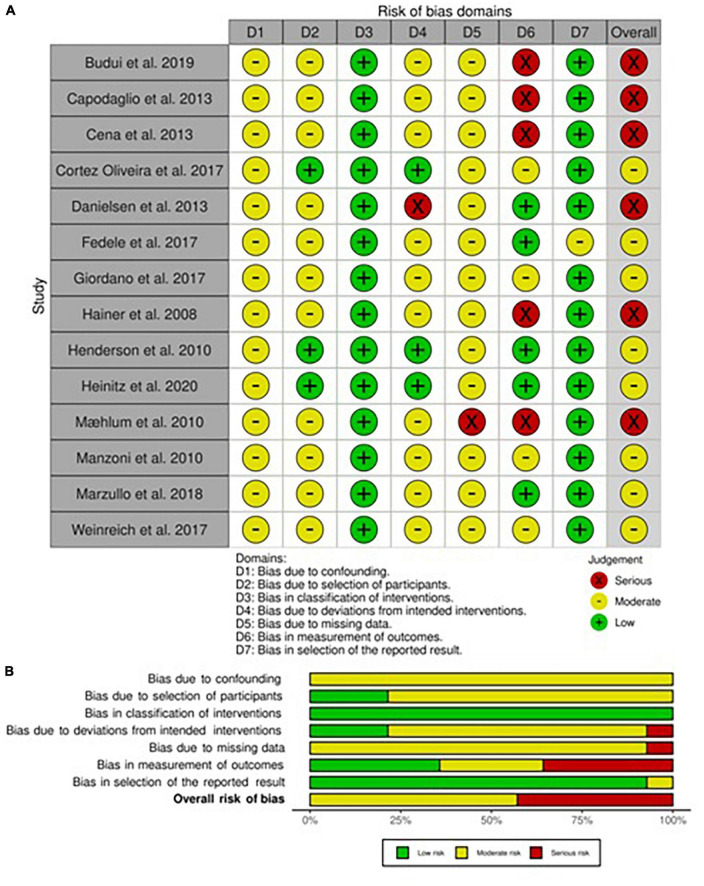
Risk of bias within longitudinal studies in adults. **(A)** Traffic light plot and **(B)** summary plot presenting the risk of bias within the longitudinal studies included in the systematic review.

**FIGURE 3:**
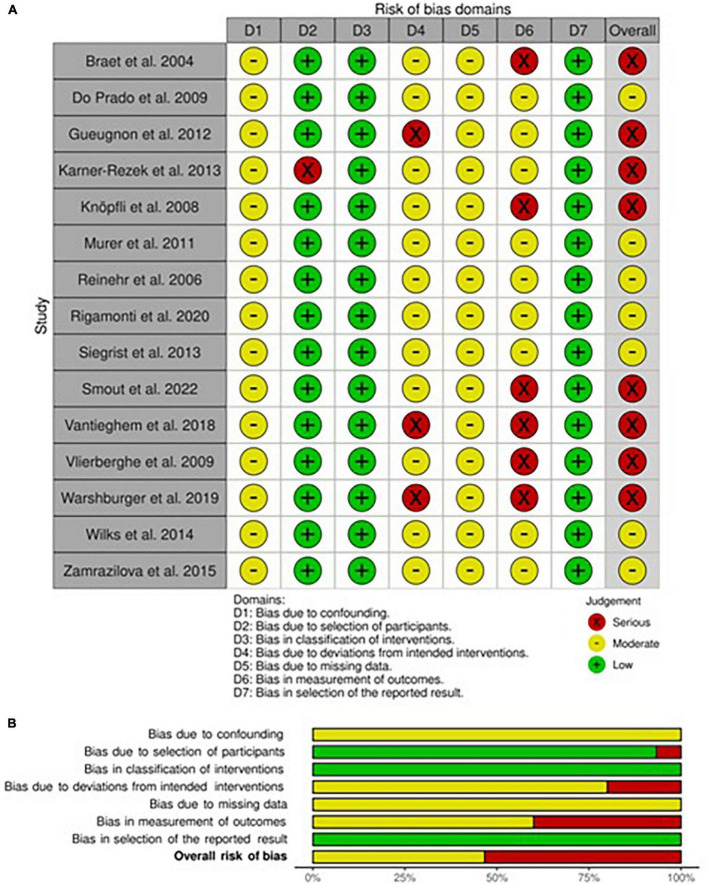
Risk of bias within longitudinal studies in children and adolescents. **(A)** Traffic light plot and **(B)** summary plot presenting the risk of bias within the longitudinal studies included in the systematic review.

The risk of bias assessment for each non-randomized included study in adults and children/adolescents is respectively presented in Figures 2A,B, 3A,B. Both in adults and children, all studies had shown to have a moderate risk of bias due to expected confounding factors linked to study design. On the contrary, classification bias was judged almost inexistent since arms, when different, were always well-identified *a priori*.

#### Adults

In adults, the risk of bias for the selection of participants was mainly considered moderate since most of the participants have been consequently recruited except for three studies in which patients had started the intervention at the same time (34, 41, 42). The risk of bias for deviation was dependent on the variability of interventions, mainly the ones that included physical activity, which was by nature not equal in intensity for each patient. Only one study was classified as at risk of serious bias due to the presence of both *ad libitum* food consumption and physical activity training (38). The risk of bias for missing data was mainly judged as not particularly influential even if no declaration for intention to treat analysis was present in any one of the included studies, therefore the level of risk was set at moderate. Nevertheless, this risk was judged serious in a high dropout rate study (37). Finally, the risk of bias assessment in the measurement of outcomes led to a high plausible prevalence of observer bias and self-administered questionnaires, which brought to a serious risk in five studies (27, 30, 31, 37, 41) and moderate in four (24, 29, 39, 42). On the contrary, only in one study, the risk of bias for the selection of outcomes was judged moderate due to the natural poor detection from instruments used (28).

#### Children and adolescents

As regards the assessment of the risk of bias in this subcategory of studies, we evaluated that all studies had a low risk of selection bias, except one (51) that deliberately excluded possible participants without complete data and thus classified as serious bias. Also, all studies shared a moderate level of risk for bias for missing data, for the same reasons explained above. Some studies (46, 53, 58) have been judged as a possible source of deviation bias (serious risk) since no fixed diet was provided and *ad libitum* or normocaloric diets were chosen instead. The risk of bias in the measurement of outcomes was, as previously explained, substantially high in most studies (moderate risk), with six studies classified as serious risk due to the characteristics of evaluated outcomes (46, 50, 52–55). On the other hand, the risk of bias in the selection of reported results was low.

### Risk of bias within randomized controlled trials

#### Adults

**FIGURE 4:**
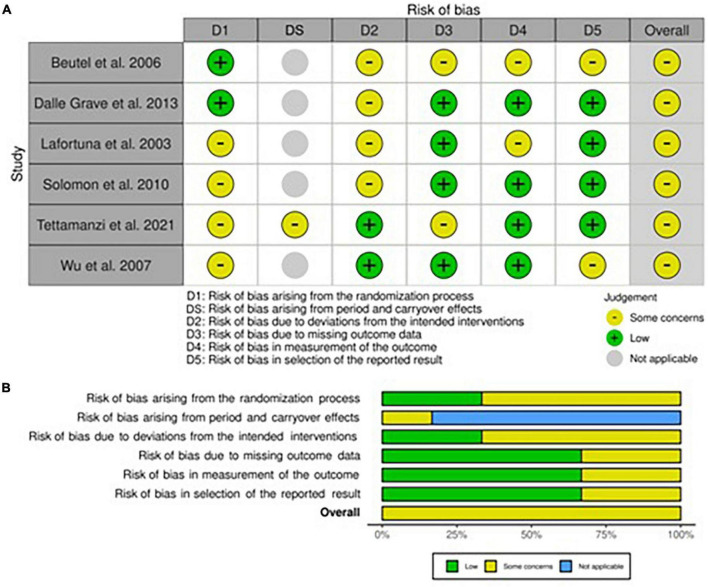
Risk of bias within Randomized Controlled Trials (RCTs) in adults. **(A)** Traffic light plot and **(B)** summary plot presenting the risk of bias within the RCTs included in the systematic review.

**FIGURE 5:**
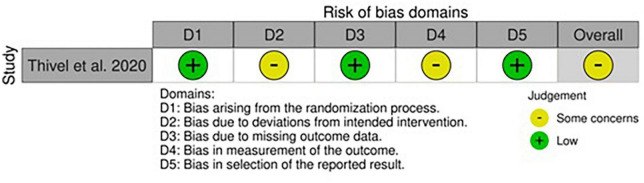
Risk of bias within RCTs in children and adolescents.

All studies showed at least one concern among the categories of bias analyzed (Figures 4A,B, 5). Some concerns for allocation were individuated in four articles due to a lack of description of randomization design (25, 33, 35, 43). As previously described, studies that included a physical activity protocol that did not specify intensity level or diet without a precise indication of calories were judged at medium risk of deviation bias (25, 32, 35, 40). Missing data were also having some concerns in two studies since the attrition analysis couldn’t still completely ensure the inexistence of bias due to a high dropout percentage (33, 40). The risk of bias for the measurement of outcomes was mainly lower compared to non-randomized studies even if some concerns were individuated in studies that included non-completely objective measurements (25, 40). The selection of the reported result brought concerns only in one study due to some possible interactions between study groups (40). As regards the only cross-over study, the lack of awash-out period led to a medium level of concern for the risk of bias even if no evidence exists in terms of possible contamination of results (33).

#### Children and adolescents

The only RCT in this category was almost free from concerns apart from the possible deviation bias induced by the nutritional intake screening tool and the risk of bias for measurement of outcomes, where some minor flaws were predicted due to the lack of blinding and personal interpretation from the authors (57).

### Dietary regimens

#### Adults

Specific characteristics of the multidisciplinary treatments in adults are resumed in Supplementary Tables 1a–3a. The moderately low-calorie diet, up to 800–1,000 kcal deficit from predicted, or calculated adequate energy intake, was by far the most used nutritional intervention among the studies, often following the national or international recommendations of a healthy and balanced diet, i.e., composed of a percentage of carbohydrates equal to 45–60% of the daily energy intake, of a 20–35% of lipids, and the remainder from proteins, generally equal to about one gram per kilogram of ideal body weight (60–62). A separate case was the study by Dalle Grave (32), in which two moderately hypocaloric and low-fat diets were tested. The findings suggested that a high-protein and hyperglucidic diet were similar in terms of changes in weight and body composition if the calorie and fat intake remain constant.

As regards the two studies in which a normocaloric diet was given, the aim of them was the comparison of two different experimental interventions, rather than the mere weight loss. In the first, Beutel et al. investigated whether there was a difference in weight loss and choice of food administered through self-service when treating participants with two psychotherapeutic interventions (behavioral and psychodynamic approaches) (40). In the second study, Solomon et al. compared two normocaloric diets with various glycemic indexes by also prescribing intense physical activity in order to achieve a calorie deficit (35).

Severe hypocaloric diets, with more than 800–1,000 kcal deficit and therefore under basal metabolism level, were administered in three studies instead (34, 36, 41).

#### Children and adolescents

Specific characteristics of the multidisciplinary treatments in adults are resumed in Supplementary Tables 1b–3b. A percentage (25%) of interventions were administering normal calorie diet regimens in association with physical activity as a weight management program (46, 53, 57, 58), while in the remaining studies moderately hypocaloric diets were given. All studies were following the recommended proportion of macronutrients for balanced diets except for two studies from Belgium (52, 54) and one study from Germany (47) in which low-fat diets were administered.

### Physical activity

#### Adults

Scheduled physical activity was part of the multidisciplinary program in 16 out of 20 studies. Aerobic physical activity was the most frequently prescribed, alone, or in association with muscle-strengthening activities. The programs of physical activity were not always specified in detail (27, 28, 39, 41). The described activities ranged from a minimum of 3 h per week (26, 40, 43) to 14 h per week (29, 31), mostly indoors, except in the two Norwegian studies (37, 38) and another one with outdoor walks programs (25). Several sports, described in detail and included in a daily program of public access, were also encouraged in the Norwegian studies.

#### Children and adolescents

Physical activity was present in all studies. Compared to studies in adulthood, exercise programs were described in a more detailed manner except in a few cases, in which it was not possible to assess the duration of the sessions (46, 53, 57). The time dedicated to the activities varied from a minimum of 5–6 h per week (44, 55, 58) to a maximum of 28 h (59). Aerobic activity was often made up of both individual sports (swimming, skiing, walking, etc.) and team sports (football, volleyball, etc.) while muscle strengthening activity was present in fewer studies (45, 49–56).

### Psychotherapy and education

#### Adults

Apart from the previously described study by Beutel et al. (40) in which the effectiveness of the psychodynamic and cognitive-behavioral approaches was compared, the latter method was certainly the most widely used in all studies that included psychotherapy as a part of the multidisciplinary rehabilitation program. The cognitive-behavioral technique was mainly used in individual sessions (24–27, 29–31, 40, 41), but also group sessions were fairly present (24, 29, 31, 32, 37–40). Globally, about two-thirds of all adult studies included psychotherapy.

Educational sessions were concerning healthy eating lessons or dietary consultations and were present in less than half of the studies (24–26, 30, 32, 37–39).

#### Children and adolescents

Cognitive-behavioral approach was applied individually in five studies (47, 48, 55, 56, 58), as like in groups (45, 46, 48, 52, 54). As opposed to adults, psychotherapy had, in some cases, also been addressed to the families of children and adolescents (44, 47, 48, 55). The sessions dedicated to psychotherapy were often less described compared to adult studies.

Education not only included nutritional lessons (45, 46, 48, 55, 56, 58) but also physical activity and behavioral techniques for patients (45, 46). Dietetic, cooking, or behavioral approach lessons were provided also for parents in two cases (44, 48). In two other studies, patients were able to attend to a special school service associated with the center (52, 54).

### Adherence

#### Adults

The dropout rate was recorded in five studies (33, 35–37, 40). In the other studies, we could assume that in RCT all participants concluded the intervention while in longitudinal studies no evidence exists to discriminate between no-dropout, or no-intention to treat analysis. The dropout rate was generally low except for the study by Maehlum et al. where at the of the study about 40% of subjects were lost (37).

#### Children and adolescents

In the only RCT the dropout rate was three out of 24 patients (57). The remaining studies did not provide any information on dropout or whether the intention to treat analysis was done.

### Efficacy

#### Adults

Data from analyzed studies (Supplementary Table 3a) have shown that inpatient rehabilitation always provided a body weight reduction, ranging from 3% in 3 weeks to 13% in 12 weeks. Body composition data was provided in several studies but a high heterogeneity was observed between the parameters used. Body fat variation ranged from −3 to −26%, while fat-free mass loss was up to 9% compared to baseline. This translates into a reduction in percentage fat mass that did not exceed 6%, while in two cases body fat% resulted to increase after treatment (28, 36). As regards the variation of other secondary outcomes, it was registered a maximum reduction of mean SBP by 13% and mean DBP by 10%, blood glucose (between –5 and –10%), total cholesterol (from −12– to −16%) and triglycerides (−0/−32%). HDL cholesterol ranged from −9% (31) to + 13% (28) after treatment. In three studies (all Italian), an increase in uric acid levels by 2–7% was registered after hospitalization (28, 29, 31).

In terms of specific design, RCT studies were generally aimed to investigate specific areas of nutrition or multidisciplinary approaches rather than reinforcing a standardized protocol. One study compared behavioral and psychodynamic approaches during weight loss, claiming that behavioral looks more effective but only in terms of maintaining the weight at follow-up (40). Two studies tested two different diets in each of the arms keeping the other interventions constant. In the first one, it is claimed that the content of carbohydrates and proteins in a three-week diet does not influence weight loss provided that the amount of fat and energy is kept constant (32). In the second one, a low glycemic index diet lowered more significantly hyperglycemia and hyperinsulinemia compared to a high glycemic index diet (35). Another cross-sectional study comparing a high protein diet and a Mediterranean diet found that the first dietary intervention appears more effective in reducing insulin resistance within 21 days of diet (33). Finally, one study investigated whether there was a difference between non-specific training and individualized training concluding that they brought the same loss of weight but individualized training was more effective in preventing weight regain at follow-up (25).

Some of the longitudinal studies were focusing on publishing the results of a consolidated weight management rehabilitation program without adding anything novel (29, 31, 37–39), while sometimes they were introducing a particular outcome of interest as the obesity-related disability (30), uncoupling proteins expression (42), natriuretic peptide level (28), leptin (34, 41), psycho-behavioral factors influence on weight loss (41), tissue remodeling (34), metabolic response (36), quality of life (24), and resting energy expenditure as predictors of weight loss (26).

#### Children and adolescents

In pediatrics (Supplementary Table 3b), inpatient treatments had a longer duration but body weight reduction was more relevant, ranging between 4% in 3 weeks and 52% in 10 months. The sources that provided data on body composition also showed that the total fat mass had been lowered on average by 6–35%, while the lean tissue had decreased by 1–7%, translating into a relative change in fat mass% that was always negative, up to a maximum of –31%. Data on blood parameters was less present, with a documented reduction of total cholesterol by 13–14% in two studies (45, 47) and by 28% in another one (49) while triglycerides decreased by 8% (47) and 43% (49) in two studies, and slightly increased in other two (45, 48). Blood pressure variation was reported in three studies (47–49), with a decrease of 4–7% for SBP and 4–9% for DBP.

Going more specifically into treatment achievements, the only RCT evaluated whether there was a difference between food preferences at *ad libitum* buffets when providing two different kinds of physical activity, concluding that eccentric cycling showed a greater improvement in body weight, body composition and a less willing of fat foods compared to concentric cycling (57).

Regarding longitudinal trials, also in this case, some of them were not focused on specific aims, providing only generic data on treatment outcomes (44, 50, 52), while most were also focusing on specific aspects such as the variations of adiponectin or leptin (48, 49, 58), physical performance (51), comparison with outpatient intervention (47), presence of metabolic syndrome (56), fatigue (53, 55), cognitive functions (53), or presence of cognitive disorders (54).

### Follow-up data

#### Adults

Beutel et al. showed that one year after discharge, about 90% of patients returned for the follow-up visit but 60% of them gained weight since discharge and 24% of this percentage returned with a body weight higher than when she was hospitalized (40). In another Norwegian study, it was found that at the first follow-up (6 months) almost all patients lost more weight than at discharge, while at 12 months a good percentage of patients regained weight, even if none had returned to pre-admission weight (38). Lafortuna et al. (25) as previously described, showed that existed a difference, even if not statistically significant, between non-specific training and individualized training in terms of weight regain at follow-up.

#### Children and adolescents

Siegrist et al., reporting the follow-up data after one year, showed that about 50% of the boys did not return for the follow-up visit, while the remaining half lost further weight (48). In another study, after 10 months of hospitalization and 14 months of follow-up, 44% of children and adolescents were still overweight and, on average, about 75% of the weight lost during hospitalization was recovered between discharge and follow-up visit (52). Finally, Warschburger et al. showed that after 6 months the overall trend was of further weight loss, but with 30% of adolescents who had worsened compared to discharge. At the following follow-up (12 months), also in this case the result was that of a partial recovery of the lost weight (46).

## Discussion

Lifestyle intervention is widely recognized as the most efficacious, safe, and cost-effective strategy for all stages of the prevention of obesity and its complications (11, 63–66). Despite being such an important and mandatory instrument, its effect has proven to be greatly influenced by several modifiable and non-modifiable factors that also involve social, cultural, economic, and psychological aspects. Nevertheless, an increase in compliance has been shown as multidisciplinary programs include different non-dietetic interventions such as behavioral therapy, which, on its own, improves both diet and physical activity adherence (67, 68).

As widely shown in this review, the effectiveness of inpatient weight reduction programs seems to be undisputed in the short term, heavily impacting body weight, cardiovascular and other risk factors associated with obesity, and can be mainly explained by compliance (51). In the long term, the lack of follow-up data and the unpromising results claimed by some of the authors in the subsequent visits raise the evidence that something is still lacking in the management of these kinds of interventions, with possible causes outlined below.

This is the first review in literature that provides a clear and well-rounded description of current practice in rehabilitative weight management programs specifically aimed at weight loss, thus excluding articles in which the inpatient obesity treatment was secondary to another hospitalization reason or acute illness such as in another recent work by Seida et al. In this review, authors performed a research focusing on the evidence on rehabilitation for hospitalized patients with obesity. In that case, a total of 39 studies were included, with one overlapping study with this review (24), but with a completely different set of assumptions, since most of the included articles were concerning post-acute rehabilitation that also aimed at weight loss, but the latter not being the first criteria for the treatment (9).

The data we gathered is indicating that in the short term the efficacy of the modification of weight, cardiovascular risk factors and body composition is undisputed and is generically higher than outpatient lifestyle intervention. To make a comparison, a meta-analysis by Hassan et al. reported that obese adults who were provided a lifestyle intervention can achieve a BMI reduction by −0.3 to −4.0 kg/m^2^ in the range of 3–24 months (69), while Galani et al. in 2007 concluded that the difference in means compared to controls is –5.1 kg achieved in up to 3 years (70).

In obese children, the cumulated results on the efficacy of an up to 2 years lifestyle intervention are resumed in a meta-analysis by Mandy et al., for a reduction in BMI by −1.25 kg/m^2^ (71).

In our review, even if it was not possible to perform a meta-analysis, we can assume that the weight loss achieved is higher due to a greater consistency (51). In other words, the probability of losing weight after an inpatient intervention is much higher than outpatient lifestyle interventions, but the pace of losing weight is about the same if the compliance with these treatments is maximum. Still, it is hard to compare the two categories since the duration of interventions is quite different from each other.

Along with body weight changes, body composition remodeling has been assessed in about 40% of included studies, even if it was not homogeneously described within them. In adults, the loss of relative fat mass percentage was low and although it was not possible to evaluate whether the presence of physical activity was associated with lean mass preservation during weight loss, it is plausible that studies that included scheduled physical activity, especially resistance training, were the ones that had the best results in terms of body composition improvement, as showed by recent evidence (72, 73). At the same time, a severe hypocaloric diet was associated with a high loss in lean mass. On the other hand, obese adults have a higher risk of occurring in acute complications due to high-intensity physical activity, also as a consequence of previous and coexisting cardiovascular, musculoskeletal and respiratory chronic disease, which makes it harder to schedule higher intensity or frequency. On the contrary, in pediatrics, the preservation of fat-free mass was higher, and this can be mainly explained both by the different anabolic predisposition of the body to synthesize muscle proteins and the higher capability to perform sports and high-intensity training despite the increased weight (74–77).

Another interesting point in this matter is also that a good percentage of analyzed rehabilitation programs for children and adolescents were characterized by a normal calorie diet in association with physical activity, even without specific research purposes. This is because children and adolescents, especially those under the age of 16 years, require a substantial supply of nutrients which should not normally be reduced to ensure correct growth whether it be from a physical, hormonal, or mental point of view. As a consequence, all recent guidelines agree with recommending low-calorie diets only in cases of advanced obesity or failure of all previous attempts to regain normal weight due to possible deficiencies in micronutrients. In addition, physical activity in children and adolescents represents a positive reinforcement rather than a deprivation and should be always encouraged as a correct lifestyle teaching (78–80).

Among the change in biological parameters, a curious trend was pointed out in three Italian studies, reporting an increase in uric acid (28, 29, 31), despite weight loss and a contemporary reduction in triglycerides, except in one. This finding has raised the hypothesis that food patterns in the prescribed diets were responsible for this increase. Since it was difficult to assess whether the weekly menu of each study was following a more Mediterranean or animal source pattern, because the description of each diet was scarce, we can only hypothesize that hyperuricemia was caused by an excess of purines from meat and fish, and a shortage of low-fat cheeses and legumes which should be prevalent in a classic Mediterranean diet (81). Furthermore, this aspect points out that some concerns could exist with hospital menus, which are often not consistently assessed for nutritional adequacy and patient satisfaction (82, 83). For this reason, even if no definitive data regarding food variety is available in this setting of care, we assume that some improvements could be made, also in to increase the nutritional knowledge of patients.

Another issue found within investigated articles involves two studies carried out in the same center in Belgium (52, 54) where minors were treated for 10 months with a moderately low-calorie diet but largely reduced in lipids (about 40 g per day and 14% of daily calories). Since the authors did not justify the choice within the article, there is a small possibility that it was given misleading information regarding the nutritional program used, also because no evidence exists supporting the administration of diets with a percentage of fat under 20% of daily calories (84).

Finally, an important aspect of a multidisciplinary intervention is psychological support. Although research shows the importance of the effectiveness of psychological treatment for obese patients, especially CBT, the percentage of those who receive proper treatment for this disorder outside hospitalized care is low (85). Therefore, this aspect acquires even more relevance in this setting of care, since the patients are given the best environment to start focusing on themselves, if they have not already started, contemporarily assisted by a multidisciplinary team. Indeed, behavioral psychotherapy has the fundamental role of educating patients to understand the modern obesogenic environment they are living in, which includes high-stress levels, lack of time for cooking and family, abundant presence of cheap, highly palatable, energy-dense foods and the induced sedentary lifestyle (86, 87). Furthermore, the relationship between mental health and obesity involves several diseases and pathological mechanisms such as depression, addictions, attention deficit disorder, and binge eating disorder, which alone was shown to have a prevalence of around 40–50% in this population (88, 89). For many people, eating is also the easiest way of coping with stress, anxiety, isolation, abuse, despair, and frustration (86). For these reasons, it appears intuitive that psychotherapy should be present in every obesity rehabilitation setting as a way to increase the chances of long-term weight loss. Fundamentally tied to these aspects and complicating them further, the stigma of obesity has proven to be present in all contexts of care, with evidence that health professionals themselves could demonstrate implicit and explicit weight bias toward people living with overweight or obesity, even those that specifically treat these patients (90). Accordingly, it has been pointed out that weight stigma should be considered a modifiable psychosocial risk factor that could be targeted as part of treatment efforts during and after inpatient weight loss programs. Recent evidence has in fact suggested that addressing weight stigma in clinical treatment may improve emotional well-being and health behaviors, with patients themselves asking for ways to cope with these experiences (91). On the other hand, healthcare professionals have an important role to play by engaging in supportive, non-stigmatizing communication, both toward adults and youths (90, 92). Among pediatrics, we have evidence in literature showing that family-based behavioral treatment appears useful in some cases, although in studies in adolescents it has been less successful. This is partly explained by the fact that some of the eating disorders linked to obesity start to grow in severity during adolescence, thus the cognitive-behavioral treatment among pediatrics should be personalized depending on age (93). On the other hand, the consequences of stigma and weight bias could start very early in childhood, together with a higher risk of depression, anxiety, low self-esteem, and body dissatisfaction that must be addressed as soon as possible, with rehabilitation settings being a crucial and realistic solution for these cases (94).

### Limitations

This review brings some limitations. First, only studies written in English were included, thus a publication bias might be present. Also, the overall quality extracted through the bias evaluation tools claims a moderate degree of uncertainty in the interpretation of results. Included studies’ designs were quite heterogeneous in terms of exercise intensity, energy provided in diets, presence of educational and behavioral sessions, and study length. Finally, despite the relative importance of short-term results, as widely stated throughout the work, this systematic review only focused on these data; therefore, the speculation on long-term effects is based on scarce evidence and needs further studies, especially RCT, which were lacking in this review.

### Conclusion

In conclusion, despite the other limitations that may affect its overall evidence, the heterogeneity of studies was highly representative of the current practice in this narrow and evolving area, which is by its nature highly split up among countries and continents. Sufficient evidence exists to affirm that inpatient weight loss programs are consistently efficient toward amelioration of health outcomes in the short term: not only body weight but also a beneficial modification of quality of life, psychological well-being, eating behavior, physical performance, and fatigue are associated with this treatment. Inpatient programs should be considered as an alternative choice in case of patients looking for a more structured intervention, without a whole set of impairments that may affect outpatient treatments, or simply for subjects not willing to undergo surgery or take additional drug therapies. In addition, in pediatrics, these programs have been gradually rising in importance, also as an educational instrument and as a potential tool to prevent chronic obesity and eating disorders in adulthood.

To keep pace with innovation in pharmacotherapy and surgery, however, inpatient weight loss programs could still largely benefit in long-term efficacy by accurate planning both on a national and an international level. Particularly in the psychological and educational area, there is a critical demand for evidence on the relationship between different approaches and follow-up effects. More studies focusing on personalization and innovative rehabilitation designs are therefore necessary to further enhance the knowledge in this field, potentially leading to a completely new dimension of research in the treatment of obesity.

## Data availability statement

The original contributions presented in this study are included in the article/Supplementary material, further inquiries can be directed to the corresponding author.

## Author contributions

DS designed this review. DS, FG, ST, VA, and FP participated in the first draft. DS, SG, and VA cooperated toward the screening of literature and the quality assessment. TD, AA, GA, PM, and MC contributed to the manuscript review and editing. All authors were involved in the final approval of the submitted version.
