# Supplementary material for: Beyond bariatric surgery and weight loss medicaments. A systematic review of the current practice in obesity rehabilitative inpatient programs in adults and pediatrics
Source: Front Nutr. 2022 Sep 29;9:963709. doi: 10.3389/fnut.2022.963709 (PMC9556721; doi:10.3389/fnut.2022.963709)
Supplement: Supplementary file 1 [file Data_Sheet_1.docx]

***Supplementary Material***

Table S1a. Characteristics of inpatient RCT studies in adults

| **First author, year** | **Country** | **Setting** | **Inclusion criteria** | **Exclusion criteria** | **Dietary intervention** | **Non – dietary parallel treatments** | **Number of subjects (M-F)** | **Intervention duration** | **Outcomes** | **N° of arms** | **Summary** |
| --- | --- | --- | --- | --- | --- | --- | --- | --- | --- | --- | --- |
| **Beutel et al. 2006** | GERMANY | Rehabilitation clinic | BMI ≥ 35 plus psychiatric morbidity | / | Regular meals with self-service | Behavioral approach or Psychodynamic approach, + group therapy, relaxation, physical training, nutritional education | 154 D.O: 24 +  168 D.O:  21 | 50.4 day/ 47.4 days | Body weight, distress, systolic and diastolic pressure | 2 | Behavioral and Psychodynamic approach are comparable in terms of weight loss and distress but behavioral is more effective looking at follow up in terms of maintaining the weight loss |
| **Dalle Grave et al. 2013** | ITALY | Weight disorder inpatient unit | BMI > 40  Or BMI > 35 and < 40 with at least one weight loss-responsive comorbidity (e.g., type 2 diabetes,  cardiovascular diseases, sleep apnea, severe joint disease, two or  more risk factors defined by the Adult Treatment Panel III | Pregnant or lactating, took medications  affecting body weight, had medical comorbidities associated  with weight loss or had severe psychiatric disorders (e.g., acute psychotic  states, bipolar disorder, bulimia nervosa). | High protein diet (HPD)  34%  PRO, 46% CHO, 20% LIP.  1500 kcal for men, 1200 kcal for women or high carbohydrates diet (HCD).  17% PRO, 63% CHO, 20% FAT.  1500 kcal for men, 1200 kcal for women | Cognitive behavioral therapy, educational sessions, physical activity (6 days/week 30 min aerobic plus 2 days/week 30 min calisthenics) | 43  (17-26)  +  45  (20-25) | 3 weeks | BMI, (body weight),  attrition rates, changes  in cardiovascular risk factors and psychological profile | 2 | In the short terms, the content of carbohydrates and proteins in the diet  does not influence  weight loss when the amount of fat  and energy are kept constant |
| **Lafortuna et al. 2003** | ITALY | Multidisciplinary Rehabilitation Unit | BMI ≥ 30 | Acute cardiovascular, orthopedic or neuromuscular diseases. Systematic physical activity before admission | 1200-1800 kcal/day (Basal energy expenditure – 500 kcal) 21% PROT, 53% CHO,  26% FAT | Nutritional education, cognitive-behavioural psychological counseling  + exercise training 5 days/week 30 min aerobic and 30 min resistance plus 50-70 min of leisure walking 2 days/week, resistance training  Or  individualized training | 15 (6-9) +  15 (6-9) | 3 weeks | Body weight, aerobic performance, muscle strength, locomotor capabilities | 2 | Non specific training and Individualized training bring the same result in terms of weight loss but individualized training offers better results in terms of muscle performance and physical fitness. Also, at follow-up, subjects in the individualized training group regained less weight |
| **Solomon et al. 2010** | USA | Clinical Research Unit | Age ≥ 65, BMI > 30, prediabetes, post menopausal women, weight stable for  at least the previous 6 months | Heart, kidney, liver, thyroid, intestinal,  and pulmonary diseases; medications known  to affect the outcome variables of the study or hormone replacement therapy | Low glycaemic index healthy normocaloric diet  (resting metabolic rate multiplied by a sedentary activity  factor of 1.2)  or  High glycaemic index healthy normocaloric diet  (REE multiplied by a sedentary activity  factor of 1.2 | Aerobic exercise (60 min/day 5 day/week) | 12 (3-7) D.O: 2  +  12 (5-7) | 12 weeks | Body weight, body composition, blood pressure, glycaemic profile, lipid profile, aerobic capacity | 2 | Both low and high glycemic index diets improved body composition and insulin resistance, but the latter impaired pancreatic b cell function while the low GI diet decreased both hyperglycemia and hyperinsulinemia by an higher extent relieving β cell stress |
| **Tettamanzi et al. 2021** | ITALY | Rehabilitation hospital | Women aged 20-57, BMI 35-64, HOMA-IR ≥ 3, able to perform physical activity and not following any specific dietary pattern in the 6 months preceding study enrollment | Diabetes mellitus, binge eating disorder, proton pump inhibitors, antibiotics, metformin or probiotics. | 2 hypocaloric diets (REE * 1.2 -500 kcal/die). Mediterranean (M): 55% CHO, 25% FAT and 20% PROT or high protein (HP) diet: 40% CHO, 30% FAT and 30% PROT | / | HP-M 11 (0-11) M-HP 6 (0-6)  D.O: 1 | 21 days | Body weight, body circumferences, HOMA-IR, blood pressure, lipid profile, microbiota diversity | 2 | HP diet appears to be more effective in reducing insulin resistance compared to M diet in both instances of the crossover and 10 microbes were indentified to be different between the two diets |
| **Wu et al. 2007** | TAIWAN | University Hospital | Diagnosis of schizophrenia, at least 300 mg of clozapine orally taken/day for at least a year, BMI > 27 kg/m2 | Any other antipsychotic treatment, lipid-lowering medications, ambulatory disfunctions, organ failure, acute or severe mental illness, mental retardation, pregnant, lactating women | 1,300 to 1,500 kcal per day for  women and to 1,600 to 1,800 kcal  per day for men including up to 7/8 servings of fruit and vegetables per day (55%CHO, 25% FAT, 20% PROT) | Walking for 1.62 km for  about 40 minutes and walking up 231  stairs (14 cm per stair) and down 330  stairs (13.5 cm per stair) for about 20  minutes under supervision (3 days/week) | 28 (11-17) | 6 months | anthropometric  and biochemical parameters  (serum glucose, triglyceride, cholesterol,  insulin, cortisol, prolactin,  growth hormone, IGF-1, IGFBP-3) | 2 | Intervention  significantly decreased  neuroleptic-related side effects, body weight, BMI, waist  and hip circumference, triglycerides,  insulin, and IGFBP-3 level |

*BW: Body weight; BMI: Body Mass Index; CHO: dietary Carbohydrates; PROT: dietary Proteins; FAT: dietary Fats; D.O.: Drop-out; /: no information*

Table S1b. Characteristics of RCT studies in children and adolescents

| **First author, year** | **Country** | **Setting** | **Inclusion criteria** | **Exclusion criteria** | **Dietary intervention** | **Non – dietary parallel treatments** | **Number of subjects (M-F)** | **Intervention duration** | **Outcomes** | **N° of arms** | **Summary** |
| --- | --- | --- | --- | --- | --- | --- | --- | --- | --- | --- | --- |
| **Thivel et al. 2020** | FRANCE | Pediatric obesity center | Age ≥ 12 and ≤16, BMI > 90^th^ percentile and obesity according to international cut-off points | Medications that could affect energy metabolism, tobacco or alcohol consumers, contraindications to exercise | Balanced normocaloric diet based on *ad libitum* buffet intake | Eccentric cycling or Concentric cycling | 12 (6-6) D.O: 2  +  12 (6-6)  D.O: 1 | 12 weeks | Body weight, body composition, food intake, food desire | 2 | Eccentric cycling showed a greater improvement in body weight, body composition and a less willing of fat foods with a consequent reduced energy intake compared to concentric cycling |

*BW: Body weight; BMI: Body Mass Index; CHO: dietary Carbohydrates; PROT: dietary Proteins; FAT: dietary Fats; D.O.: Drop-out; /: no information*

Table S2a. Characteristics of inpatient longitudinal studies in adults

| **First author, year** | **Country** | **Setting** | **Inclusion criteria** | **Exclusion criteria** | **Dietary intervention** | **Non – dietary parallel treatments** | **Number of subjects (M-F)** | **Intervention**  **Duration** | **Outcomes** | **N° of arms** | **Summary** |
| --- | --- | --- | --- | --- | --- | --- | --- | --- | --- | --- | --- |
| **Budui et al. 2019** | ITALY | Multidimensional rehabilitation program clinic | Severe obesity and obesity-related comorbidities | Patients with a major orthopedic, neurologic, or psychiatric disease, as well as  those with a clinical indication to use a very low-calorie diet (i.e., prior to bariatric, orthopedic or cardiac surgery) | Caloric deficit of  800–1,000 kcal/day with respect to the estimated individual daily total energy requirements. PROT 0.8-1g kg/ideal BW. CHO 50-55% FAT 25-30% | Daily educational meetings, individual and group sessions with psychologists (behavioral), 2 h of exercise per day, both aerobic and muscle strength | <65 years: 215 (70-145)  >65 years: 44 (16-28) | 3 weeks | Body weight, lipid profile, glycaemic profile, blood pressure, uric acid, kidney status, circumferences, aerobic capacity, muscle strength, quality of life | 2 | Multidimensional therapeutic program including diet, exercise, and behavioral therapy is very effective on a clinical and functional point of view in both young and older adults |
| **Capodaglio et al. 2013** | ITALY | Multidisciplinary Rehabilitation Unit | BMI > 30 plus orthopedic conditions, caucasic, able to read and write | Acute cardiovascular, respiratory,  neurological, and psychological conditions | 1200-1800 kcal/day (75% of resting metabolic rate) 21% proteins, 53% carbohydrates,  26% lipids | Nutritional education, Psychological counseling (cognitive-behavioral), aerobic exercise 45 min/day, 5 days/week.  Resistance exercise 45 min/day | 464 (92-372) | 4 weeks | Body weight, body  mass index, motor and cognitive Functional Independence Measure, Obesity-related disability test, Visual Analogue Score for functioning,  Timed-Up-Go | 1 | Multidisciplinary  intensive rehabilitation is effective in minimizing  the obesity-related disability and enhancing functional  capacities in patients with obesity-related orthopedic  conditions |
| **Cena et al. 2013** | ITALY | psycho-nutritional obesity rehabilitation structure | Both sexes, age >20 and <60, BMI ≥ 30 | Existing psychiatric pathologies or previously diagnosed psychological disorders, endocrinological pathologies that affect weight trends,  assumption of drugs/medications that alter body weight, denial of informed consent to be evaluated | Balanced diet with an average energy value of 7,535 kJ | Individualized physical activity (7 days/week)(not specified), psychological interviews (not specified) | 42 (13-29) | 3 weeks | Body weight, body circumferences, psychological well-being, body uneasiness, depression, binge behavior, other psychometric tests | 1 | No significant difference between weight losses In the underreporter and non-underreporter  subgroups. Women achieved a better improvement from a psychological point of view, while men from a physical one |
| **Cortez Oliveira et al. 2017** | BRAZIL | University Hospital | BMI ≥ 40 | Cancer, psychiatric disorders, thyroid disorders, pregnant, lactating, women with history of alcohol or tobacco | 1800 kcal the first day, 1500 the second, 1200 from the third till the end of the study; 50-60% CHO, 20-30% FAT, 15% PROT | / | 11 | 6 weeks | Body weight, body composition, resting metabolic rate, body circumferences, glycaemic profile, lipid profile, | 1 | Loss of body weight, fat mass and fat free mass. UCP2 expression is directly associated with %weight loss |
| **Danielsen et al. 2013** | NORWAY | Obesity Clinic | BMI ≥ 40 or BMI ≥35 with comorbidities, able to walk for at least 20 min | Pregnancy, participation in previous treatments at the clinic | Breakfast and lunch: buffet with restricted portion sizes (1900 kcal total for men and 1600 kcal for women). 50-60% CHO, <30% FAT, 20% PROT | Training sessions (aerobic, strength, agility), sport activities and walking tours (at least 90 min/day); Dietary consultations; behavioral group session therapy | 71 (29-42) | 10-14 weeks (mean = 12) | Body weight, body composition, body circumferences, glycaemic profile, lipid profile | 2 | Reduction of prevalence of metabolic syndrome, favorable changes in cardiovascular disease risk factors. At 12 months, most of the subjects did not regain weight |
| **Fedele et al. 2017** | ITALY | Multidisciplinary Rehabilitation Unit | BMI ≥ 30 | Hypertension, diabetes, chronic heart failure, cardiac valvulopathy, cirrhosis, chronic obstructive sleep disease, cancer, eGFR < 60 ml/min/1.73m^2^ | Hypocaloric diet, not specified | Personalized physical activities | 14 (4-10) | 3 weeks | Body weight, body composition, body circumferences, glycaemic profile, lipid profile, blood pressure, uric acid, β-type natriuretric peptide | 2 | Weight reduction is associated with a significant increase of β-type natriuretric peptide |
| **Giordano et al. 2017** | ITALY | Rehabilitation clinic | Overweight or obese | / | 1200 to 1500 kcal/day for females and from  1200 to 1800 kcal/day for males; CHO 50-55%, FAT 25-30%, PROT 0,8-1g/kg ideal weight | Lifestyle and nutritional education meetings, individual and group behavioral psychological therapy; 120 min/day of physical activity (aerobic and resistance training) | 136 (53-83) | 3 weeks | Body weight, body circumferences, glycaemic profile, lipid profile, blood pressure, quality of life, physical performance | 1 | Multidimensional  short-term program induces  multiple improvements in domains in severe obesity subjects |
| **Hainer et al. 2008** | CZECH REPUBLIC | Weight management spa | Women who exhibited stable  weight on a 7 MJ/day diet during the 1st week of weight  management | Endocrine disorders, type 2 diabetes, medications that affect body weight | 4.5 MJ/day = 1075 kcal/day (PROT 26.0 %, FAT 28.0 %, CHO 46 %) | Daily physical activity (not specified) and cognitive behavioral modification of the  lifestyle | 67 (0-67) | 3 weeks | Body weight, body circumferences, body composition, glycaemic profile, lipid profile, psycho-behavioral tests, hormones | 1 | Weight loss is associated with hormones levels, age, BMI and C reactive protein at baseline, while psycho-behavioral factors do not contribute to weight change |
| **Henderson et al. 2010** | USA | General Clinical Research Center, University of Vermont | Class 1 and Class 2-3 obese women | Not taking medications  known to affect metabolism, waist circumference <88cm | 900-1350 Kcal/day  (50%CHO, 30% FAT, 20% PROT) | / | 12 (0-12) | 30 days | Body weight, body circumferences, body composition, glycaemic profile, metabolic rate, β-hydroxy butyrate, free fatty acids, plasma amino acids, hormones | 1 | Caloric restriction impairs tissue remodeling; the more obese individuals  experienced more rapid tissue remodeling |
| **Heinitz et al. 2020** | USA | Clinical Research Unit | Overweight (BMI ≥27) and obese volunteers without comorbidities | / | Subject specific liquid diet, 50% energy compared to a weight maintaining diet, (58% CHO, 27%FAT, 15% PROT) | / | 11  D.O: 1 | 6 weeks | Body weight, body composition, bomb calorimetry (food oxidation), energy expenditure, hormones | 1 | Less metabolic slowing induced by caloric restriction is associated with greater longer-term loss of weight. Metabolic response to diet is individually variable and can be seen at the very first week of diet |
| **Mæhlum et al. 2010** | NORWAY | Obesity Clinic | BMI ≥ 40 or BMI ≥35 with comorbidities, able to walk for at least 20 min | / | Low calorie diet  according to the National Standards in Norway | Healthy cooking training, nutritional lectures, Two to three training sessions  each of which lasts for 45 min (bicycling, Nordic pole walking, dance, strength training, yoga, pilates, sports) | 166 D.O: 60 (not specified) | 14 weeks | Body weight, blood pressure, lipid profile, glycaemic profile, liver profile, quality of life | 1 | Almost completely  untrained obese people can manage an aggressive physical  training programs with two to three sessions every day and if the physical activity is vary they can also enjoy it |
| **Manzoni et al. 2011** | ITALY | Cardiovascular rehabilitation Unit | BMI ≥ 30 and cardiovascular disease (history of myocardial infarction, coronary  artery by-pass grafting, coronary angioplasty) | Recent  (less than four weeks) myocardial infarction, coronary  artery by-pass grafting or coronary angioplasty | 80% of resting energy expenditure (50% of energy from CHO, 30% FAT, and 20% PROT) | Nutritional counseling, cognitive-behavioural psychological counseling, exercise sessions (6 days/week)of aerobic activity | 176 (113-63) | 4 weeks | Body weight, exercise capacity, psychological well-being | 1 | Multi-dimensional rehabilitation program reduces body weight, increases exercise capacity and improves psychological and emotional well-being. Well-being was found to be associated with weigh loss |
| **Marzullo et al. 2018** | ITALY | Multidisciplinary Rehabilitation Unit | Severily obese (BMI ≥ 40), euthyroid patients  Stable weight in the prior three months | Pharmacological therapies,  cardiac disorders, peripheral edematous congestion, cardiac symptoms, endocrine obesity, autoimmune or  chronic inflammatory disorders, type 1 and 2 diabetes mellitus, chronic  obstructive pulmonary disease, history of neoplasms or degenerative diseases, previous  chronic steroid treatment, kidney disorders, and liver disease | 75% of resting energy expenditure (52% of energy from CHO, 30% from FAT, and 18% from PROT) | Psychological and nutritional counseling (not specified),  aerobic physical  activity program, 60 min/day of cycle ergometer pedaling, treadmill  walking and stationery rowing (5 days/week), | 100 (50-50) | 4 weeks | Body weight, body circumferences, body composition, thyroid hormones, resting energy expenditure | 1 | High baseline resting energy expenditure is a predictor of its large reduction during a weight loss program; fat free mass, thyroid hormones, insulin, leptin and sympathetic tone are all associated with energy expenditure also in a short term period of time |
| **Weinreich et al. 2017** | GERMANY | Metabolical rehabilitation Center | BMI > 30 with no physical limitations of the musculoskeletal system, inappropriate exhaustion during ordinary physical stress or cardiac/pulmonary limitations | Severe acute cardiopulmonary pre-existing diseases, severe accompanying diseases (tumour diseases), decompensated heart, failure, fatigue, rhythm disturbances, dyspnea or angina pectoris during ordinary physical stress, severe orthopedic disease, addiction (except nicotine abuse), patients undergone bariatric surgery, pregnancy, and secondary obesity | Hypocaloric mixed diet with a reduced fat content. 500-800 kcal of energy deficit, but not inferior to 1200 kcal/day | Behavioral therapy  (group sessions),  physical fitness (not specified), nutritional counseling | 52 (40-12) | 4 weeks | Body weight, blood pressure, heart rate, glycaemic profile, body composition, quality of life | 1 | An inpatient rehabilitation program is necessary to achieve an initial weight reduction and an improvement of physiological state and life quality in patients for whom outpatient therapy is not suitable since a lifestyle change is required |

*BW: Body weight; BMI: Body Mass Index; CHO: dietary Carbohydrates; PROT: dietary Proteins; FAT: dietary Fats; D.O.: Drop-out; /: no information*

Table S2b. Characteristics of longitudinal studies in children and adolescents

| **First author, year** | **Country** | **Setting** | **Inclusion criteria** | **Exclusion criteria** | **Dietary intervention** | **Non – dietary parallel treatments** | **N° of subjects (M-F)** | **Intervention Duration** | **Outcomes** | **N° of arms** | **Summary** |
| --- | --- | --- | --- | --- | --- | --- | --- | --- | --- | --- | --- |
| **Braet et al. 2004** | BELGIUM | Obesity treatment center | Age from 7 to 17 years, overweight, defined as BMI > 95^th^ percentile, outpatient treatment failed | Mental deficiency | Hypocaloric diet 1,400 to 1,600 cal per day (53% CHO, 14% FAT, 33% PROT) | Sport events (10 hours/week) plus 4 hours/week of individual exercises as cycling, swimming, jogging, or abdominal exercises; cognitive-behavioral treatment in small groups only for the first 4 months | 122 (not specified) | 10 months | Body weight, height, psychological well-being, parental report, eating disorders | 1 | Younger children loss higher amounts of weight in percentage; at 14 month follow up 44% of children were still overweight; a 10 month intervention may be unnecessary to achieve these results |
| **Do Prado et al. 2009** | GERMANY | Rehabilitation Center | BMI > 99.5^th^ percentile and metabolic disorders (hyperinsulinemia, diabetes  mellitus type 2, hyperlipidemia, hyperuricemia) and orthopedic problems, psychological disturbances following extreme obesity, plus motivation for long-term therapy | Untreated psychiatric disorders,  lack of motivation and obesity syndromes requiring  permanent control | Hypocaloric diet (1500/1800 kcal) with high vitamin and fiber content, 52% CHO,  30% FAT, and 18% PROT | Dietetic and cooking education, individualized physical exercise (swimming, hiking, biking, skiing, lifting training; 90 min, 4 days/week); family-oriented psychotherapy approach with elements of behavior, analytic and relaxation methods | 688 (235-453) | 6,3 months | Body weight, height, body composition | 1 | Multidisciplinary therapy allows significant reduction  in severe obesity, preserving growth and percentage  of fat free mass. At similar obesity levels and age ranges, an  additional favorable effect is found in boys |
| **Gueugnon et al. 2012** | FRANCE | Childhood obesity center | BMI > 97^th^ French percentile | Systemic diseases, endocrine disorders, syndromic obesity | Balanced diet, 2300-2500 Kcal/day (56% CHO,  30% FAT, and 14% PROT) | Nutritional education, physical activity (walking, ball games, swimming or climbing, 45/60 min, 5 days/week), individualized behavioral therapy and psychological care | 32 (10-22) | 9 months | Body weight, height, glycaemic profile, adiponectin, resistin | 1 | Determination of adiponectin isoforms could be more useful than measurement of total adiponectin for enhancing therapeutic intervention programs |
| **Karner-Rezek et al. 2013** | SWITZERLAND | Children hospital | Children and adolescents who provided a full set of data, overweight or obese based on BMI | Secondary obesity and underlying endocrine diseases | Children/adolescents of 50 kg to 80 kg received ∼1400 kcal/day and subjects over 80 kg received ∼1600 kcal/day (55-60% CHO, 25-30% FAT, 15-20% PROT) | Individually adapted physical activity (2 session of 60-90 minutes of endurance/aerobic type activities (walks, basketball, hockey, soccer, handball, swimming, water games, skiing, snowboarding) plus a 4-5 hours hiking/snow walking once a week plus strength training | 28 (19-9) | 8 weeks | Body weight, height, body composition, physical performance, muscle strength | 1 | Subjects in good anaerobic fitness were more likely to lose weight and fat mass and to preserve lean body mass; decrease in body mass and fat mass were neither associated with the over­all energy expenditure nor with the energy deficit |
| **Knöpfli et al. 2008** | SWITZERLAND | Children hospital | Severely obese children based on BMI | Same as Karner-Rezek | Children less than 50 kg received ∼1200 kcal/day, 50 kg to 80 kg received ∼1400 kcal/day, over 80 kg received ∼1600 kcal/day (55-60% CHO, 25-30% FAT, 15-20% PROT) | Same as Karner-Rezek | 128 (77-51) | 8 weeks | Body weight, height, body composition, physical performance, muscle strength, quality of life | 1 | Boys lost more weight and body fat; improvement in the image and perception of one’s own body may result in a better control of pleasant and healthy lifestyle behaviors |
| **Murer et al. 2011** | SWITZERLAND | Children hospital | Body mass index > 98th  percentile for age and sex | Secondary  obesity, underlying  endocrine diseases, type 2 diabetes, impaired glucose  tolerance, or other major medical problems | Same as Knöpfli | Same as Knöpfli | 203 (114-89) | 8 weeks | Body weight, height, body composition, glycaemic profile, lipid profile, blood pressure, leptin | 1 | High baseline leptin concentrations  in obese children and adolescents may not necessarily reflect  leptin resistance and leptin levels greatly decrease during the weight loss |
| **Reinehr et al. 2006** | GERMANY | Children hospital | Age ≥ 10 and ≤ 14, obese children according to international task force of obesity in childhood | Endocrine disorders or syndromic obesity | Mixed diet with 1030 Kcal/day, 56% CHO, 18% FAT, 26% PROT | Physical exercise (1-2 hours/day)  consisting of jogging, ball games, and swimming, nutrition  education, and behavioural therapy both on children and their families | 119 (60-59) | 6 weeks | Body weight, height, glycaemic profile, lipid profile, blood pressure | 2 | A short term inpatient intervention (6 weeks) is comparable with a long term outpatient intervention (1 year) in terms of weight loss; the first better improved total and LDL cholesterol, while the second better increased HDL and reduced insulin and triglycerides |
| **Rigamonti et al. 2020** | ITALY | Auxology Division | Children and adolescents with BMI ≥ 95^th^ percentile from age and sex specific Italian charts | Physical inability or cognitive impairments | 1200-1700 kcal/die (75% of REE), 21% PROT, 53% CHO, 26% FAT | Nutritional education (every day), psychological counseling (2-3 times/week), physical activity (5 hours/week) including indoor light jogging, dynamic exercises for upper and lower limbs, aerobic exercises and outdoor walking | 548 (236-312) + 96 (43-53) | 3 weeks | Body weight, lipid profile, glycaemic profile, blood pressure, balance (OLSB), climbing test score (SCT), fatigue (FSS) | 2 | The beneficial effects of the multidisciplinary program were not different among patients with or without metabolic syndrome |
| **Siegrist et al. 2013** | GERMANY | Pediatric rehabilitation clinic | Age ≥ 6 and ≤ 19, overweight (BMI > 90^th^ percentile) or obese (BMI > 97^th^ percentile) | Endocrine disorders or sindromal obesity, unwilling or unable to complete at least 4 weeks | Optimized balanced diet with energy intake based on height and sex (from 1250 to 1800 kcal) 55% CHO, 30% FAT, 15% PROT | Theoretical and practical lessons of healthy eating, physical activity (10 hours/week of sports plus 6 hours/week of walks or excursions), cognitive-behavioral therapy, supportive training to parents | 402 (164-238) | 5,5 weeks | Body weight, height, lipid profile, glycaemic profile, blood pressure, leptin, adiponectin | 1 | Baseline BMI predicted weight loss better than baseline leptin; half of subjects were lost at follow-up, while the remaining lost additional weight |
| **Smout et al. 2022** | ITALY | Auxology Division | Age 11-18, BMI-SDS ≥ 2 according to italian growth charts | Same as Rigamonti | 1200-1700 kcal/die (75% of REE), 21% PROT, 53% CHO, 26% FAT | Nutritional education (every day), psychological counseling for children (2-3 times/week) and parents (1 times/week), physical activity (5 hours/week) including indoor light jogging, dynamic exercises for upper and lower limbs, aerobic exercises and outdoor walking | 100 (36-64) | 3 weeks | Body weight, BMI-SDS, fatigue (PedsQL-MFS), behavioral questionnaires (YSR) | 1 | Body weight, BMI and fatigue all decreased after the intervention. Fatigue reliably changed only in a small portion of the sample. |
| **Vantieghem et al. 2018** | BELGIUM | Obese children Residential center | Obese adolescents, Age ≥ 12 and ≤ 18 | / | From 1450 to 2690 kcal/day  51% CHO, 23% PROT, 26% FAT | Individualized  and monitored exercise (strength, endurance swimming), physiotherapy (psychomotor training), | 48 (12-36) | 30 weeks | Body weight, height, body composition, self-perceived fatigue, cognitive performance | 1 | Adolescents had positive changes in body composition,  cognitive improvement and decreased self-perceived fatigue; greater fat mass loss  was associated with improved cognitive functions |
| **Vlierberghe et al. 2009** | BELGIUM | Obesity treatment centre | Age ≥ 14 and ≤ 18, overweight, defined as BMI > 95^th^ percentile | Learning disability, presence of developmental syndromes | 1,400 to 1,600 cal per day (53% CHO, 14% FAT, 33% PROT) | Sport events (10 hours/week) plus 4 hours/week of individual exercises as cycling, swimming, jogging, or abdominal exercises; cognitive-behavioral treatment in small groups only for the first 3 months | 66 (22-44) | 10 months | Body weight, height, psychological disorders, eating disorders | 1 | The presence of at least  one psychological disorder appeared a negative predictor of weight loss; All eating disorders resolved, but a substantial number of adolescents still suffered from psychological disorders at the end of the treatment |
| **Warshburger et al. 2019** | GERMANY | Rehabilitation clinics | Age ≥ 16 and ≤ 21, obese (BMI > 97^th^ percentile or > 30) plus at least a comorbidity | Inadequate language skills, severe cognitive impairments, secondary obesity | *Ad libitum* | Aspecific lifestyle intervention, nutritional counseling, physical activity program (not specified), cognitive-behavioral therapy  or As previous plus an age-specific group intervention program with topics such as diet, eating behaviors, stress managements, problem-solving, interaction with parents, school and profession, social competence | 125 (48-77) + 141 (44-97) | 5,6 weeks | Body weight, height, quality of life | 2 | Age-sensitive approach is not superior to an approach that address a wider age- range in terms of weight loss and quality of life; a slight reverse of the achievement reached was observed from 6 month to 12 month follow-ups |
| **Wilks et al. 2014** | GERMANY | Weight loss clinic | 6 to 19 years old  BMI >90^th^ percentile | Secondary obesity, syndromal disorders or monogenetic diseases | Optimized balanced diet  (30% FAT, 15% PROT 55% CHO), energy intake of 1200–  1800 kcal/day, depending on body height and sex | 11 hours/week of  structured and supervised moderate-intensity physical  activity (swimming,  hiking, group sports as well as strength and posture  training), group behavioral therapy and healthy eating lessons | 429  (169 – 260) | 5,4 weeks | Body weight, height, body circumferences, heart rate recovery, glycaemic profile, lipid profile | 1 | Heart rate recovery, body weight and cardiovascular risk factors significantly improved; heart rate recovery was not correlated with body weight or cardiovascular risk factors but was associated with parasympathetic function |
| **Zamrazilova et al. 2015** | CZECH REPUBLIC | Weight management specialized center | Obese girls, BMI ≥ 97^th^ percentile for sex and age of the Czech reference | / | Hypocaloric diet, 5–7 MJ/day according to  age and BMI (50-55% CHO, 30% FAT, 15-20% PROT, sugar ≤ 5%) | Aerobic exercise 3.5/4.0 hours/day (jumping, jogging, ball games, fitness, swimming, dancing and hiking 4–10 km) | 184 (0- 184) | 4 weeks | Body weight, height, body circumferences, skinfolds, body composition, glycaemic profile, lipid profile, bood pressure, liver profile, c-reactive protein, hormones (adiponectin, resistin, leptin, ghrelin, glp-1, adipsin, visfatin), presence of Adenovirus 36 antibodies | 1 | No differences found in self-reported dietary intake  between Adenovirus 36 antibody positive and negative obese; energy restriction in antibody positive girls was  associated with greater decrease of abdominal obesity and preservation of subcutaneous adipose tissue than in those who were antibody negative |

*BW: Body weight; BMI: Body Mass Index; CHO: dietary Carbohydrates; PROT: dietary Proteins; FAT: dietary Fats; D.O.: Drop-out; /: no information*

Table S3a. Resume of multidisciplinary characteristics and impact on outcomes in adult studies

| **First author, year** | **Dietary Intervention** | | **Physical activity (hours or sessions/week)** | | | **Psychological (hours or sessions/week) and educational interventions** | | **Duration** | | **Mean ± standard deviation**  **or median baseline values** | | | **End of treatment mean ± standard deviation or Δchange from baseline ± standard deviation**  **(+ percentage change from baseline)** | | |
| --- | --- | --- | --- | --- | --- | --- | --- | --- | --- | --- | --- | --- | --- | --- | --- |
| **Della Grave et al 2013** | High Protein Diet (HPD) | High CHO Diet (HCD) | Aerobic (3 hours/week)  Calisthenics (1 hour/week) | | | CBT in groups (5 session/week)  +  Educational sessions (2 sessions/week) | | 3 weeks | | HPD:  BMI: 45.8 ± 6.5  SBP: 139.9 ± 21.3 DBP: 85.5 ± 10.9 | | HCD:  BMI: 45.4 ± 7.0  SBP: 140.9 ± 18.8  DBP: 82.7 ± 8.9 | HPD:  Weight: -5.9 ± 2.7  BMI: -2.0 ± 0.8 (-4.4%)  SBP: -16.6 ± 16.7 (-11.9%)  DBP: -9.1 ± 9.1 (-10.6%) | HCD:  Weight: -5.5 ± 2.3  BMI: -1.9 ± 0.8 (-4.2%)  SBP: -19.1 ± 16.3(-13.5%)  DBP: -5.7 ± 8.9 (- 6.9%) | |
| **Beutel et al. 2006** | *Ad libitum* | | Generic (1 session/week), Swimming (1.2 sessions/week), Ergometer (1 session/week) | | Generic (2.6 session/week), Swimming (0.6 sessions/week), Ergometer (0.7 session/week) | Psychodynamic  Individual therapy (2 sessions/week), group sessions 1.5 sessions/week) | CBT individual therapy (1.6 sessions/week), group sessions (3 sessions/week) | 47.4 days | 50.4 days | BMI: 43.9 | | BMI: 44.6 | Weight: -4.39 ± 2.6% | Weight: -4.58 ± 2.8% | |
| **Budui et al. 2019** | Balanced hypocaloric diet | | Both aerobic and resistance training (14 hours/week) | | | Individual therapy (1 session/week),  Group therapy (2 sessions/week) | | 3 weeks | | Youngs:  Weight: 121.4 ± 29.2  BMI 43.9 ± 9.4  Glycemia: 101.3 ±29.7  Cholesterol: 182.7±34.4  HDL: 48.2±15.2  Triglycerides: 129.3± 62.4  Uric acid (mmol/L): 6.5 ± 1.5  SBP: 130.2±19.0  DBP: 77.9±11.0 | | Old:  Weight: 112±21.6  BMI: 43.9 ± 4.2  Glycemia: 104.6±23.8  Cholesterol: 179.3±37.4  HDL: 50.5±17.7  Triglycerides: 113.9±55.7  Uric acid: 6.8 ± 1.7  SBP: 133.2±17.2  DBP: 78.3±12.1 | Youngs:  Weight: -4.3 ± 1.8%  BMI: -4.4 ±2.2 %  Glycaemia: -8.1 ±12.9 %  Cholesterol: -15.0 ± 12.6 %  HDL: -9.2 ± 15.8 % Triglycerides: -10.5 ± 26.4%  Uric acid: +4.5 ± 22.1%  SBP: -5.4 ± 11.1 %  DBP: -5.0 ± 13.0 % | Old:  Weight:: -3.9 ± 1.7%  BMI: -3.9 ±2.2 %  Glycaemia: -8.3 ±13.5 %  Cholesterol: -14.0 ± 14.1 %  HDL: -9.3 ± 16.0 %  Triglycerides: -0.8 ± 36.2%  Uric acid: +2.8 ± 18.5%  SBP: -7.2 ± 9.4 %  DBP: -7.2 ± 9.4 % | |
| **Capodaglio 2013** | Balanced moderately hypocaloric diet | | Aerobic activity (from 2,5 to 5 hours/week) plus resistance training (from 3,5 to 7 hours/week) | | | CBT individual therapy (2 sessions/week)  +  Nutritional education | | 4 weeks | | BMI: 41.61 ± 6.06 | | | Weight: from -4.97 % to -4.04 % depending on disability | | |
| **Cena 2013** | Balanced moderately hypocaloric diet | | Individualized physical activity (7 days/week)(not specified) | | | psychological interviews (not specified) | | 3 weeks | | Males: BMI: 48 ± 12.3  Females: BMI: 40.4 ± 7.0 | | | Males: BMI: -2.1 (-4.3%)  Females: BMI: -1.3 (-3.2%) | | |
| **Cortez Oliveira et al. 2017** | Balanced hypocaloric diet | | / | | | / | | 6 weeks | | Weight: 155  BMI: 58.5  FFM: 65.4  FM: 89.5  Glycemia: 95.7  Cholesterol: 180.3  Triglycerides: 145 | | | Weight: 146.5 ( -5.5%)  BMI 55.3 (-5.5%)  FFM 63.1 (-3.51%)  FM 83.4 (-6.8%)  Glycemia 85.7 (-10.4%)  Cholesterol 152.6 (-15%)  Triglycerides 126.3 (-12.9%) | | |
| **Danielsen et al . 2013** | Balanced moderately hypocaloric diet | | Training sessions (aerobic, strength, agility), sport activities and walking tours (from 7,5 hours/week to 11,25 hours/week) | | | Behavioral group session therapy (not specified)  +  Dietary consultations | | 10/14 (mean 12) weeks | | Weight: 128.9 ± 19.4  BMI: 42.8 ± 4.6  FM: 60.5 ± 11.0  FFM: 68.3 ± 13.2 | | | Weight: -17.5 ± 6.1 (-13.6%)  BMI: -5.8 ± 1.8 (  FM: -15.7 ± 5.3 (-25.9%)  FFM: -1.7± 2.5 (-2.5%) | | |
| **Fedele et al. 2017** | Hypocaloric diet, not specified | | Personalized physical activity (not specified) | | | / | | 3 weeks | | Weight: 118.6 ± 24.1  BMI: 42.3 ± 1.6  SBP: 119.3 ± 3.8  DBP: 74.3 ± 3.7  FM%: 51.3 ± 1.9  FFM%: 48.8 ± 7.6  Glycemia: 96.4 ± 7.0  Cholesterol: 191.1 ± 45.3  HDL: 44.4 ± 12.3  Triglycerides: 122.4 ± 56.2  Uric acid: 5.7 ± 1.1 | | | Weight: 114.5 ± 23.8(-3.5%)  BMI: 40.8 ± 1.6 (-3.5%)  SBP 118.6 ± 3.4 (-0.5%  DBP 77.8 ± 3.0 + (3%)  FM %: 52.2 ± 1.6 (+1.7%)  FFM% = 48.4 ± 7.5 (-0.8%)  Glycemia: 86.8 ± 2.7 (– 10%)  Cholesterol 165.1 ± 45.3 (-13.6%)  HDL: 50.2 ± 15 (+ 13%)  Triglycerides: 102-8 ± 46.3 (– 15.9%)  Uric acid: 5.9 ± 1.2 (+3.5%) | | |
| **Giordano 2017** | Balanced moderately hypocaloric diet | | Both aerobic and resistance training (14 hours/week) | | | Individual therapy (1 session/week),  Group therapy (2 sessions/week) | | 3 weeks | | Weight: 120.4 ± 26.0  BMI: 43.2 ± 7.4  Glycemia (mmol/L): 5.74 ± 1.64  Cholesterol (mmol/L): 4.75 ± 1.06  HDL (mmol/L): 1.20 ± 0.38  Triglycerides (mmol/L): 1.46 ± 0.72  Uric acid (μmol/L): 455.0 ± 99.2  SBP: 139.4 ± 19.3  DBP: 82.6 ± 11.8 | | | Weight – 3.9 ± 1.6 %  BMI -3.9 ± 1.6 %  Glycemia -7.0 ± 15.1 %  Cholesterol -15.9 ± 12.3 %  HDL -6.5 ± 14.4 %  Triglycerides -9.7 ± 24.8 %  Uric acid + 7.5 ± 21.0%  SBP – 9.8 ± 12.3 %  DBP -9.7 ± 13.8 % | | |
| **Hainer et al. 2008** | Hypocaloric and moderately high protein diet | | Daily physical activity (not specified) | | | Behavioral therapy (not specified) | | 3 weeks | | Weight: 84.6 ± 12.9  BMI: 32.4 ± 4.5  FM%: 41.7 ± 5.7  FFM: 48.8 ± 4.7  Glycemia (mmol/L): 5.1 ± 1.6  Leptin: 21.2 ± 9.1 | | | Weight 3.80 ± 1.64 (- 4.5%)  BMI: -1.5 ± 0.63 (-4.6%)  FM% -2.7 ±2.9 (-6.5%)  FFM -0.3 ± 3.2 (-0.6%)  Glycaemia -0.3 ± 1.7 (-5.8 %)  Leptin: -5.9 ± 6.5 (– 27.8 %) | | |
| **Henderson et al. 2010** | Balanced hypocaloric diet | | / | | | / | | 30 days | | Moderately obese:  Weight: 78.1 ± 3.2  BMI: 31.2 ± 0.2  FM%: 37.0 ± 1.3  FFM: 49.3 ± 2.4 Leptin:29.6 ± 3.5 | Severely obese:  Weight: 115.0 ± 9.0  BMI: 42.5 ± 2.7  FM% 42.1 ± 1.0  FFM: 66.4 ± 4.6  Leptin 40.7 ± 4.9 | | Moderately obese:  Weight: 73.1 ± 2.9 (-6.4%)  BMI: 29.2 ± 0.3 (-6.4%)  FM% 35.4 ± 1.0 (-4.3%)  FFM: 47.2 ± 1.8 (-4.3%)  Leptin: 11.3 ± 1.7 (-61.8%) | Severely obese:  Weight: 107.2 ± 7.6 (-6.7 %)  BMI: 39.6 ± 2.4 (-6.7%)  FM%: 41.6 ± 0.9 (-1.2%)  FFM: 62.4 ± 4.0 (-6%)  Leptin : 30.1 ± 7.0 (-26%) | |
| **Heinitz et al. 2020** | Balanced hypocaloric liquid diet | | / | | | / | | 3 weeks | | Weight: 108.6 ± 20  BMI: 38.9 ± 6.7  FM%: 62.6 ± 4.5  FFM: 68.4 ± 15.4 | | | Weight: 98.4 ± 16 (-9.4%)  BMI: 35.4 ± 5.5 (-9.0%)  FM%: 62.7 ± 5.6 (+1.5%)  FFM: 61.9 ± 12.5 (-9.5 %) | | |
| **Wu et al. 2007** | Balanced moderately hypocaloric diet | | Walking and walking up stairs (3 hours/week) | | | / | | 6 months | | Weight: 78.4 ± 11.6  BMI: 30.43 ± 4.2  FM%: 36.9 ± 7.8 | | | Weight: -4.2 ± 4.4 (-5.4 %)  BMI: -1.59 ± 1.66 (-5.2%  FM%: -1.3 ± 6.4 (-3.5%) | | |
| **Lafortuna et al. 2003** | Balanced moderately hypocaloric diet | | Non specific training:  Combination of aerobic, walking and resistance training (7,5 hours/week) | Individualized training:  Cycloergometer (2,5 hours/week) and strength training (not specified) | | Individual behavioral therapy or cognitive behavioral strategies (2-3 sessions/week)  +  Nutritional education | | 3 weeks | | Weight: 115.1 ± 13.8  BMI: 40.0 ± 4.7 | Weight: 111.4 ± 14.6  BMI: 40.4 ± 3.3 | | Weight: -4.3 ± 1.1 % | Weight: -4.2 ± 1.2% | |
| **Maelum et al. 2011** | Balanced moderately hypocaloric diet | | Training sessions (aerobic, strength, agility), sport activities and walking tours (from 7,5 hours/week to 11,25 hours/week) | | | Behavioral group session therapy (not specified)  +  Dietary consultations | | 14 weeks | | Weight: 136 ± 22.7  BMI 45.7 ± 8.6 | | | Weight: 119.5 ± 23.5 (-12.1%) | | |
| **Manzoni et al. 2011** | Balanced moderately hypocaloric diet | | Cycloergometer (3 hours/week) and walking (4,5 hours/week) | | | Individual consultations (2 sessions/week) and group sessions (1 hour/week)  +  Nutritional counseling | | 4 weeks | | Weight: 106.2 ± 17.6  BMI: 38.1 ± 5.1  METs: 5.59 ± 2.52 | | | Weight: -3.9 ± 1.8 (-3.7%)  BMI: -1.38 ± 0.6 (-3.6%)  METs: 6.76 ± 2.57 (+20.9%) | | |
| **Marzullo et al. 2018** | Balanced moderately hypocaloric diet | | Aerobic activity (3 hours/week) | | | Psychological counseling (not specified)  +  Nutritional counseling | | 4 weeks | | BMI: 45.1 ± 4.8  FM%: 46.7 ± 6.8  FFM: 67.2 ± 13 | | | BMI: -5.5 ± 1.8%  FM%: -1.6 ± 6.7%  FFM: -3.7 ± 7.7% | | |
| **Solomon et al. 2010** | Low glycemic index normocaloric diet (LoGIX) | High glycemic index  normocaloric diet (HiGIX) | Aerobic activity (5 hours/week) | | | / | | 12 weeks | | LoGIX:  Weight: 97.4 ± 6 3  BMI: 34.9 ± 1.1  FM: 46.8 ± 2.0  FFM: 49.9 ± 2.9  Cholesterol: 214.6 ± 11.3  HDL: 53.3 ± 3.5  Triglycerides: 164.2 ± 26.1  SBP: 127 ± 3  DBP: 76 ± 3 | HiGIX:  Weight: 94.7 ± 4.4  BMI: 34.1 ± 1.1  FM: 42.0 ± 2.2  FFM: 55.2 ± 3.6  Cholesterol: 207.3 ± 8.3  HDL: 49.8 ± 4.1  Triglycerides: 110.9 ± 12.2  SBP: 133 ± 5  DBP: 79 ± 3 | | LoGIX:  Weight: 89.6 (– 8.0%)  BMI: 32.1 ± 1.3 (-8.0%)  FM: 40.0 (– 14.5%)  FFM: 49.7 (– 0.4%)  Cholesterol: 183.5 ± 9.6 (– 14.5%)  HDL: 50.3 ± 3.9 (-5.6%)  Triglycerides: 110.9 ± 12.2 (-32.5%)  SBP: 118 ± 2 (– 7.1%)  DBP: 73 ± 2 (–3.9%) | | HiGIX:  Weight: 85.7 ± 4.1 (-9.5%)  BMI: 30.9 ± 1.2 (-9.4%)  FM: 36.1 ± 2.9 (-14%)  FFM: 53.9 ± 3.5 (-2.3%)  Cholesterol: 181.2 ± 9.2 (-12.6%)  HDL: 47.0 ± 3.6 (-5.6%)  Triglycerides: 89.0 ± 14.2 (-19.7%)  SBP: 119 ± 4 (-10.5%)  DBP: 71 ± 3 (-7.6%) |
| **Tettamanzi et al. 2021** | Balanced moderately hypocaloric diet (M) | Moderately hypocaloric and hyperproteic diet (HP) | / | | | / | | 21 days | | HP-M  Weight: 118.9 ± 18.0  BMI: 44.6 ± 4.6  SBP: 127.3 ± 11.0  DBP: 70.1 ± 8.6  Cholesterol (mmol/L): 4.12 ± 0.78  HDL (mmol/L): 1.17 ± 0.1  Triglycerides (mmol/L): 1.27 ± 0.34  Glycemia: 93.27 ± 8.13 | M-HP  Weight: 130.9 ± 38.8  BMI: 50.4 ± 10.8  SBP: 129 ± 11.4  DBP: 79.0 ± 8.9  Cholesterol: 4.65 ± 0.86  HDL: 1.0 ± 0.1  Triglycerides: 1.6 ± 0.6  Glycemia: 94.8 ± 6.5 | | HP-M  No data available for the global treatment | | M-HP  No data available for the global treatment |
| **Weinreich et al. 2017** | Moderately hypocaloric and low fat diet | | Physical fitness (not specified) | | | Behavioral group sessions therapy  (not specified)  +  Nutritional counseling | | 4 weeks | | Weight: 135.0 ± 26.8  BMI: 43.8 ± 8.1  SBP: 135.9 ± 12.5  DBP: 84.7 ± 6.8  FM%: 38.5 ± 7.5  FFM: 83.2 ± 16.7 | | | Weight: -7.1 ± 3.4 (– 5.3%)  BMI: -2.3 ± 1.0 (-5.2%)  SBP: -12.8 ± 9.0 (– 9.4%)  DBP: -6.8 ± 5.4 (– 8%)  FM%: -2.2 ± 1.1 (– 6.1%)  FFM: -2.5 ± 4.7 (– 3%) | | |

*CHO: Carbohydrates; CBT: Cognitive-Behavioral Therapy; BMI: Body Mass Index; SBP: Systolic blood pressure; DBP: Diastolic blood pressure; HDL: High density lipoprotein cholesterol; FM: Fat Mass; FM%: Fat mass %; FFM: Fat free mass; FFM: Fat free mass%; METs: Metabolic equivalent of tasks.*

*Units of measure, if not specified in each row, are the following: Weight(kg); BMI(kg/m^2^); SBP(mmHg); DBP(mmHg); Cholesterol(mg/dL); HDL(mg/dL); Triglycerides(mg/dL); Glycemia(mg/dL); FM(kg); FFM(kg); FM%(%body weight); FFM%(%body weight); Uric acid(mg/dL); Leptin(μg/l); METs(score)*

Table S3b. Resume of multidisciplinary characteristics and impact on outcomes in pediatric studies

| **First author, year** | **Dietary Intervention** | **Physical activity (hours or sessions/week)** | | **Psychological (hours or sessions/week) and educational interventions** | | **Duration** | **Mean ± standard deviation**  **or median baseline values** | | | **End of treatment mean ± standard deviation (or median) or Δchange from baseline ± standard deviation**  **(+ percentage change from baseline)** | | |
| --- | --- | --- | --- | --- | --- | --- | --- | --- | --- | --- | --- | --- |
| **Braet et al. 2004** | Moderately hypocaloric and hypolipidic diet | Sport events (10 hours/week) and individual physical activity (4 hours/week) | | Cognitive-behavioral treatment in small groups (not specified)  +  School associated with the center | | 10 months | Weight: 84.1 ± 20.0  BMI: 32.2 ± 5.2 | | | Weight: 63.5 ± 15.2 (-24.5%)  BMI: 23.6 ± 3.9 (-26.7%) | | |
| **Do Prado et al. 2009** | Balanced moderately hypocaloric diet | Individualized physical activity (6 hours/week) | | Family oriented behavioral psychotherapy (2 weekends)  +  Dietetic and cooking education for parents | | 6,3 months | Boys:  Weight: 127.8 ± 26.4  BMI: 41.2 ± 7.1  FM%: 41.5 ± 30.2  FFM: 73.7 ± 12.7 | | Girls:  Weight: 112.3 ± 20.9  BMI: 40.0 ± 6.5  FM%: 48.1 ± 5.0  FFM: 57.5 ± 7.6 | Boys:  Weight: 100.0 ± 19.6 (-21.7%)  BMI: 31.8 ± 5.4 (-22.8%)  FM%: 30.2 ± 7.3 (-27.2%)  FFM: 69.1 ± 10.9 (-6.2%) | | Girls:  Weight: 90.6 ± 15.4 (-19.3%)  BMI: 32.3 ± 5.3 (-19.3%)  FM%: 40.3 ± 5.8 (-16.2%)  FFM: 53.5 ± 6.3 (-6.9%) |
| **Gueugnon et al. 2012** | Balanced normocaloric diet | Walking and sports (around 5 hours/week) | | Individualized behavioral therapy (not specified)  +  Nutritional education | | 9 months | Weight: 97.7 ± 2.5  BMI: 35.6 ± 0.7  Insulin: 21 ± 1.4 | | | Weight: -10.7 kg (-10.9%)  BMI: -4.7 (– 13.2%)  FM%: -31.4%  Insulin: 13.3 ± 1.2 (-36.7%) | | |
| **Karner Rezek et al. 2013** | Balanced moderately hypocaloric diet | Aerobic activities (5-7,5 hours/week), walking (4-5 hours/week), other activities (1-2 hours/week) | | / | | 8 weeks | Boys:  Weight: 92.1 ± 16.8  FM: 40.9 ± 8.5  LBM: 48.7 ± 9.3 | | Girls:  Weight: 93.7 ± 19.4  FM: 44.3 ± 13.2  LBM: 47.8 ± 8.8 | Boys:  Weight: -10.5 ± 2.3 (-11.4%)  FM: -9.4 ± 1.8 (-23.8%)  LBM: -1.2 ± 1.5 (-2.3%) | | Girls:  Weight: -10.7 ± 4.8 (-11.0%)  FM: -9.3 ± 2.6 (-21.5%)  LBM: -2.5 ± 4.5 (-4.5%) |
| **Knöpfli et al. 2008** | Balanced moderately hypocaloric diet | Aerobic activities (5-7,5 hours/week), walking (4-5 hours/week), other activities (1-2 hours/week) | | / | | 8 weeks | Weight: 89.4  BMI: 33.4  FM%: 46.6  FM: 39.9  FFM: 46.9 | | | Weight: 76.4 (-14.5%)  BMI: 28.6 (-14.4%)  FM%: 42.2 (-9.4%)  FM: 31.7 (-20.5%)  FFM: 44.1 (-6.0%) | | |
| **Murer et al. 2011** | Balanced moderately hypocaloric diet | Aerobic activities (5-7,5 hours/week), walking (4-5 hours/week), other activities (1-2 hours/week) | | / | | 8 weeks | Weight: 93.7 ± 20.5  BMI-SDS: 2.3 ± 0.3  FM%: 46.8 ± 4.7  LBM: 48.9 ± 9.9  SBP: 114.4 ± 7.7  DBP: 71.6 ± 5.7  Cholesterol: 172.0 ± 36.0  HDL: 44.0  Triglycerides: 105.0  Leptin: 29.0 | | | Weight: 79.8 ± 17.3 (-14.8%)  BMI-SDS: 1.9 ± 0.4 (-17.4%)  FM% 41.5 ± 6.1 (-8.9%)  LBM: 47.9 ± 9.7 (-2.0%)  SBP: 109.0 ± 7.1 (-4.7%)  DBP: 68.3 ± 5.4 (-4.6%)  Cholesterol: 124.4 ± 26.0 (-27.9%)  HDL: 43.0 (-2.3%)  Triglycerides: 60.0 (-42.8%)  Leptin: 7.2 (-75.2%) | | |
| **Reinehr et al. 2006** | Balanced hypocaloric diet and hypolipidic diet | Physical exercise and sports (from 7 to 14 hours/week) | | Behavioral therapy for the children (and family)(not specified) | | 6 weeks | BMI: 28.9  BMI-SDS: 2.3  Cholesterol: 174  HDL: 39  Triglycerides:101  Insulin: 17  SBP: 120  DBP: 63 | | | BMI: -3.1 (-10.7%)  BMI-SDS: -0.43 (-18.7%)  Cholesterol: 151 (-13.2%)  HDL: 40 (+2.6%)  Triglycerides: 93 (-7.9%)  Insulin: 14 (-5.9%)  SBP: 111 (-7.5%)  DBP: 57 (-9.5%) | | |
| **Rigamonti et al. 2020** | Balanced moderately hypocaloric diet | Mixed physical exercise (5 hours/week) and aerobic (1,5 hours/week) | | Behavioral psychological counseling (2-3 sessions/week)  +  Nutritional education | | 3 weeks | Without Metabolic Syndrome:  BMI: 36.3 ± 6.7  FM%: 43.8 ± 5.5  FM: 43.7 ± 8.2 | | With Metabolic Syndrome:  BMI: 38.3 ± 6.9  FM%: 43.9 ± 6.1  FM: 41.4 ± 12.0 | Without Metabolic Syndrome:  BMI: 34.8 ± 6.4 (-3.8%)  FM%: 42.7 ± 5.8 (-2.5%)  FM: 40.8 ± 7.2 (-6.6%) | | With Metabolic Syndrome:  BMI: 36.7 ± 6.6 (-4.2%)  FM%: 43.0 ± 5.0 (-2.0%)  FM: 39.0 ± 10.7 (-5.8%) |
| **Siegrist et al. 2013** | Balanced moderately hypocaloric diet | Sports (10 hours/week) and walking (6 hours/week) | | Group behavioral therapy (5 sessions(week) and individual counseling (1-3 sessions/week)  +  Nutritional lessons  and parents training | | 5,5 weeks | BMI: 33.8 ± 5.7  BMI-SDS: 2.8 ± 0.5  HDL: 50.2 ± 12.3  Triglycerides: 65.1 ± 26.3  Glycemia: 71.8 ± 8.3  SBP: 122.2 ± 13.9  DBP: 79.6 ± 9.6  Leptin: 39.5 ± 23.7 | | | BMI: 30.5 ± 5.1 (-9.7%)  BMI-SDS: 2.4 ± 0.6 (-14.3%)  HDL: 49.6 ± 12.1 (-11.9%)  Triglycerides: 71.0 ± 31.0 (+9.1%)  Glycemia: 72.6 ± 7.9 (+1.1%)  SBP: 115.6 ± 12.5 (-5.4%)  DBP: 74.9 ± 8.9 (-5.9%)  Leptin: 18.6 ± 14.0 (-52.9%) | | |
| **Smout et al. 2022** | Balanced moderately hypocaloric diet | Mixed physical exercise (5 hours/week) and aerobic (1,5 hours/week) | | Behavioral individual psychological counseling (2-3 sessions/week) and for parents (1 session/week)  +  Nutritional education | | 3 weeks | Females:  Weight: 99.0 ± 16.3  BMI-SDS: 3.0 ± 0.5 | | Males:  Weight: 114.0 ± 19.7  BMI-SDS: 3.1 ± 0.5 | Females:  Weight: 94.7 ± 15.6 (-4.3%)  BMI-SDS: 2.8 ± 0.5 (-6.7%) | | Males:  Weight: 107.9 ± 17.9 (-5.3%)  BMI-SDS: 2.8 ± 0.5 (-9.7%) |
| **Thivel et al. 2020** | Balanced normocaloric diet *ad libitum* | Eccentric cycling (ECC) (3 sessions/week) | Concentric cycling (CON) (3sessions/week) | / | | 12 weeks | ECC:  Weight: 82.8 ± 17.4  BMI: 30.8 ± 4.9  FM%: 30.3 ± 5.1  FFM: 55.2 ± 10.3 | | CON:  Weight: 75.5 ± 11.6 BMI: 29.4 ± 3.6  FM%: 31.8 ± 4.7  FFM: 49.5 ± 8.4 | ECC:  Weight: 78.8 ± 16.0 (-4.8%)  BMI: 29.0 ± 4.5 (-5.8%)  FM%: 27.5 ± 6.6 (-9.2%)  FFM: 54.6 ± 10.6 (-1.1%) | | CON:  Weight: 72.3 ± 11.8 (-4.2%)  BMI: 27.6 ± 4.0 (-6.1%)  FM%: 29.8 ± 6.3 (-6.3%)  FFM: 48.6 ± 8.4 (-1.4%) |
| **Vantieghem et al. 2018** | Balanced normocaloric diet | Physiotherapy, fitness exercise and swimming (2-3 sessions/week) | | / | | 30 weeks | Weight: 110.6 ± 24.7  BMI: 39.9 ± 20.9  FM%: 47.3 ± 20.6  FM: 53.7 ± 21.2 | | | Weight: 91.6 ± 21.2 (-17.2%)  BMI: 32.2 ± 7.1 (-19.3%)  FM%: 36.4 ± 9.5 (-23%)  FM: 34.7 ± 16.7 (-35.4%) | | |
| **Vlierberghe et al. 2009** | Moderately hypocaloric and hypolipidic diet | Sport events (10 hours/week) and individual physical activity (4 hours/week) | | Cognitive-behavioral treatment in small groups (not specified)  +  School associated with the center | | 10 months | BMI-SDS: 2.2 ± 0.3 | | | Weight: -52.5 ± 18.2% | | |
| **Warshburger et al. 2019** | *At libitum* | Physical activity program (not specified) | | Intervention group (IG):  Behavioral group interventions (not specified)  +  Special age-oriented behavioral sessions (1,5-3 hours/week)  +  Nutritional counseling | Treated as usual (TAU):  Behavioral group interventions (not specified  +  Nutritional counseling | 5,6 week | IG:  BMI-SDS: 2.8 ± 0.6 | TAU:  BMI-SDS: 2.9 ± 0.5 | | IG:  BMI-SDS: 2.5 ± 0.6 (-10.7%) | TAU:  BMI-SDS: 2.6 ± 0.5 (-10.3%) | |
| **Wilks et al. 2014** | Balanced moderately hypocaloric diet | Sports, walking and resistance training (11 hours/week) | | Behavioral group therapy (not specified)  +  practical group lessons on healthy eating and physical activity | | 5,4 weeks | Weight: 90.7 ± 22.5  BMI-SDS: 2.8 ± 0.5  Cholesterol: 155.6 ± 32.0  HDL: 50.5 ± 12.6  Triglycerides: 64.3 ± 25.9 | | | Weight: 81.9 ± 20.0 (-10%)  BMI-SDS: 2.4 ± 0.5 (-14.3%)  Cholesterol: 134.0 ± 26.5 (-13.9%)  HDL: 50.0 ± 12.2 (-1.0%)  Triglycerides: 69.4 ±29.9 (+7.9%) | | |
| **Zamrazilova et al. 2015** | Balanced moderately hypocaloric diet | Sports, aerobic exercise and walking (24,5-28 hours/week) | | Psychologist supervision (not specified) | | 4 weeks | Adv36 negative:  BMI: 32.0  BMI-SDS: 2.9  FM%: 43.6 | | Adv36 positive:  BMI: 30.6  BMI-SDS: 2.8  FM%: 40.2 | Adv36 negative:  BMI: -6.55%  BMI-SDS: -10.7%  FM%: 43.6: -5.5% | | Adv36 positive:  BMI: -6.8%  BMI-SDS: -12.7%  FM%: -5.9% |

*BMI: Body Mass Index; SBP: Systolic blood pressure; DBP: Diastolic blood pressure; HDL: High density lipoprotein cholesterol; FM: Fat Mass; FM%: Fat mass %; FFM: Fat free mass; FFM%: Fat free mass%; LBM: Lean Body Mass; BMI-SDS: Body Mass Index Standard Deviation from national standard charts; Adv36: Human Adenovirus 36.*

*Units of measure, if not specified in each row, are the following: Weight(kg); BMI(kg/m^2^); BMI-SDS(z-score); SBP(mmHg); DBP(mmHg); Cholesterol(mg/dL); HDL(mg/dL); Triglycerides(mg/dL); Glycemia(mg/dL); FM(kg); FFM(kg); FM%(%body weight); FFM%(%body weight); LBM(kg); Leptin(μg/l); Insulin (mU/L).*
